# Oxygen therapy in acute hypoxemic respiratory failure: guidelines from the SRLF-SFMU consensus conference

**DOI:** 10.1186/s13613-024-01367-2

**Published:** 2024-09-05

**Authors:** Julie Helms, Pierre Catoire, Laure Abensur Vuillaume, Héloise Bannelier, Delphine Douillet, Claire Dupuis, Laura Federici, Melissa Jezequel, Mathieu Jozwiak, Khaldoun Kuteifan, Guylaine Labro, Gwendoline Latournerie, Fabrice Michelet, Xavier Monnet, Romain Persichini, Fabien Polge, Dominique Savary, Amélie Vromant, Imane Adda, Sami Hraiech

**Affiliations:** 1 Service de Médecine Intensive-Réanimation, Nouvel Hôpital Civil, Hôpitaux Universitaires de Strasbourg, 1, Place de l’Hôpital, 67091 Strasbourg Cedex, France; 2 UMR 1260, Regenerative Nanomedicine (RNM), FMTS, INSERM (French National Institute of Health and Medical Research), Strasbourg, France; 3 Emergency Medicine Department, University Hospital of Bordeaux, 1 Place Amélie Raba Léon, 33000 Bordeaux, France; 4 SAMU57, Service d’Accueil des Urgences, Centre Hospitalier Régional Metz-Thionville, 57530 Ars-Laquenexy, France; 5 Service d’Accueil des Urgences – SMUR Hôpital Pitié Salpêtrière Assistance Publique - Hôpitaux de Paris (APHP), Paris, France; 6 Department of Emergency Medicine, University Hospital of Angers, Angers, France; 7 UNIV Angers, UMR MitoVasc CNRS 6215 INSERM 1083, Angers, France; 8 CHU Clermont-Ferrand, Service de Réanimation Médicale, Clermont-Ferrand, France; 9 Unité de Nutrition Humaine, Université Clermont Auvergne, INRAe, CRNH Auvergne, 63000 Clermont-Ferrand, France; 10 Service d’Anesthésie Réanimation, Centre Hospitalier D’Ajaccio, Ajaccio, France; 11 Unité de Soins Intensifs Cardiologiques, Hôpital de Saint Brieuc, Saint-Brieuc, France; 12 Service de Médecine Intensive Réanimation, CHU de Nice, 151 Route Saint Antoine de Ginestière, 06200 Nice, France; 13 UR2CA - Unité de Recherche Clinique Côte d’Azur, Université Côte d’Azur, Nice, France; 14 Service de Réanimation Médicale GHRMSA, 68100 Mulhouse, France; 15 Pole de Médecine d’Urgence- CHU Toulouse, Toulouse, France; 16 Université Toulouse III Paul Sabatier, Toulouse, France; 17 Service de Réanimation, Hôpital de Saint Brieuc, Saint-Brieuc, France; 18 AP-HP, Service de Médecine Intensive-Réanimation, Hôpital de Bicêtre, DMU 4 CORREVE, Inserm UMR S_999, FHU SEPSIS, CARMAS, Université Paris-Saclay, 78 Rue du Général Leclerc, 94270 Le Kremlin-Bicêtre, France; 19 Service de Réanimation et Soins Continus, CH de Saintes, Saintes, France; 20 Hôpitaux Universitaires de Paris Centre Site Cochin APHP, Paris, France; 21 Département de Médecine d’Urgences, CHU d’Angers, 4 Rue Larrey, 49100 Angers, France; 22 IRSET Institut de Recherche en Santé, Environnement et Travail/Inserm EHESP - UMR_S1085, CAPTV CDC, 49000 Angers, France; 23 Service d’Accueil des Urgences, Hôpital La Pitié Salpetrière, Paris, France; 24 Department of Research, One Clinic, Paris, France; 25 PointGyn, Paris, France; 26 Service de Médecine Intensive - Réanimation, AP-HM, Hôpital Nord, Marseille, France; 27 Faculté de Médecine, Centre d’Études et de Recherches sur les Services de Santé et Qualité de vie EA 3279, Aix-Marseille Université, 13005 Marseille, France

**Keywords:** Acute respiratory failure, Acute respiratory distress, Oxygen therapy, Conference consensus, Guidelines

## Abstract

**Introduction:**

Although largely used, the place of oxygen therapy and its devices in patients with acute hypoxemic respiratory failure (ARF) deserves to be clarified. The French Intensive Care Society (Société de Réanimation de Langue Française, SRLF) and the French Emergency Medicine Society (Société Française de Médecine d’Urgence, SFMU) organized a consensus conference on oxygen therapy in ARF (excluding acute cardiogenic pulmonary oedema and hypercapnic exacerbation of chronic obstructive diseases) in December 2023.

**Methods:**

A committee without any conflict of interest (CoI) with the subject defined 7 generic questions and drew up a list of sub questions according to the population, intervention, comparison and outcomes (PICO) model. An independent work group reviewed the literature using predefined keywords. The quality of the data was assessed using the GRADE methodology. Fifteen experts in the field from both societies proposed their own answers in a public session and answered questions from the jury (a panel of 16 critical-care and emergency medicine physicians, nurses and physiotherapists without any CoI) and the public. The jury then met alone for 48 h to write its recommendations.

**Results:**

The jury provided 22 statements answering 11 questions: in patients with ARF (1) What are the criteria for initiating oxygen therapy? (2) What are the targets of oxygen saturation? (3) What is the role of blood gas analysis? (4) When should an arterial catheter be inserted? (5) Should standard oxygen therapy, high-flow nasal cannula oxygen therapy (HFNC) or continuous positive airway pressure (CPAP) be preferred? (6) What are the indications for non-invasive ventilation (NIV)? (7) What are the indications for invasive mechanical ventilation? (8) Should awake prone position be used? (9) What is the role of physiotherapy? (10) Which criteria necessarily lead to ICU admission? (11) Which oxygenation device should be preferred for patients for whom a do-not-intubate decision has been made?

**Conclusion:**

These recommendations should optimize the use of oxygen during ARF.

**Supplementary Information:**

The online version contains supplementary material available at 10.1186/s13613-024-01367-2.

## Introduction and background

The consensus conference aims to provide evidence-based guidelines for using oxygen in hypoxemic acute respiratory failure (ARF) in adults, excluding cases related to acute lung edema and hypercapnic ARF (type II). These guidelines are intended for healthcare professionals involved in oxygen therapy in pre-hospital, hospital emergency, critical care, and intensive care settings.

## Pathophysiology key points

Hypoxemic ARF occurs when the respiratory system suddenly fails to ensure adequate oxygenation, leading to severe acute hypoxemia without hypercapnia. It is diagnosed in the absence of underlying lung disease or acute cardiogenic pulmonary edema [1], with pneumonia being the main cause.

The definition of hypoxemic ARF remains unclear, and establishing a new definition is the focus of this consensus conference. The severity of hypoxemia varies across studies, typically defined by a PaO_2_/FiO_2_ ratio of ≤ 200 mmHg or ≤ 300 mmHg. Hypoxemia results from reduced oxygen pressure in inspired air, alveolar hypoventilation, impaired alveolar oxygen diffusion, shunt, and poor ventilation-perfusion ratios.

Oxygen therapy aims to treat hypoxemic hypoxia by increasing the fraction of inspired oxygen, thereby raising arterial oxygen content. When oxygen saturation is already normal (above 96–98%), the hemoglobin’s affinity for oxygen is low, making the impact of oxygen therapy on arterial content minimal, primarily increasing PaO_2_.

Oxygen therapy can be combined with positive pressure therapy (applying super-atmospheric pressure in the airways to improve alveolar recruitment and maintain airway patency) or ventilation (applying variable pressure in the airways to assist ventilatory effort).

However, hyperoxia, defined as an excessive PaO_2_ level, can cause specific lesions, including pulmonary edema, atelectasis, retinopathy, and direct cerebral toxicity. Due to the lack of evidence defining a threshold for hyperoxia-induced damage, a PaO_2_ threshold of 100–120 mmHg has been adopted by the authors.

### Definition of oxygen therapy methods and devices

Oxygen therapy can be delivered by several devices, including conventional oxygenation, high-flow nasal cannula oxygen therapy (HFNC), positive pressure therapy and non-invasive ventilation (NIV) [2].

Conventional oxygenation employs fixed-flow devices such as nasal cannulas, single masks, Venturi masks, and high-concentration (reserve) masks. HFNC saturates the inspiratory flow with humidified, warmed air, offering several benefits: FiO_2_ stability (as the high flow rate prevents ambient air inhalation), clearance of dead space in the upper airways, reduced bronchoconstriction, improved pulmonary secretion clearance, and a limited PEEP effect, achievable only when the mouth is closed.

Several devices are designed to increase airway pressure, either continuously (Continuous Positive Airway Pressure, CPAP) or during exhalation (Positive End Expiratory Pressure, PEEP). Non-invasive ventilation (NIV) is administered with inspiratory assistance (IA) and PEEP, using a mask or helmet.

### Impact of the COVID-19 outbreak on oxygen therapy management

The COVID-19 pandemic significantly altered the use of non-invasive oxygen support, adapting to available resources and guidelines. While conventional oxygen therapy remained the most common support, the use of HFNC increased substantially, reaching 19% in a cohort of 4,643 patients admitted to intensive care for COVID-19 in France, Belgium, and Switzerland [3].

However, due to conflicting guidelines, limited evidence on device effectiveness, and concerns about aerosolization risk, the use of devices varied considerably between countries. Several simulation studies later demonstrated that the risk of aerosolization was not higher with HFNC compared to NIV or conventional oxygenation devices [4, 5].

## Methods

The « Société de Réanimation de Langue Française (SRLF)» and the « Société Française de Médecine d'Urgence (SFMU)» mandated the « Commission des Référentiels et de l'Évaluation (CRE)» and the « Commission de Référentiels (CR)» to carry out a consensus conference. The members of the two commissions defined six generic questions, and PICO (Patient, Intervention, Control, Outcome) questions [6] were then submitted to the experts (Appendix 1). An expert was appointed for each generic question proposed. An independent group of intensivists carried out the literature research. GRADE (Grade of Recommendation Assessment, Development and Evaluation) tables presenting literature data were provided [7] (Appendix 2). A level of evidence was defined for each bibliographic reference cited, depending on the type of study. This level of evidence could be re-evaluated (discounted/overvaluated) considering the methodological quality of the study. The bibliographical references common to each judgement criterion were then collected. An overall level of evidence was determined for each criterion, considering the level of evidence of each bibliographic reference, the consistency of results between the different studies, the directness of the evidence, and cost analysis. A “high” quality of evidence led to a “strong” recommendation (should, should not… GRADE 1 + or 1−). A moderate, low or very low quality of evidence led to an “optional” recommendation (probably should, probably should not… GRADE 2 + or 2−). In the absence of evidence, the issue was recommended in the form of an expert opinion. Where the literature was non-existent, the question could be the subject of a recommendation in the form of an opinion from the members of the panel. The panel was made up of 14 members, coordinated by two chairmen. All practiced in intensive care or emergency medicine. They were chosen by the organizers on the one hand for their clinical interest in the topic, and on the other because they had no related potential conflicts of interest. At the end of the conference, the role of the panel was to provide a consensus text with the conclusions and recommendations of the conference in the form of a clear answer to each of the questions. The experts wrote a text for the panel members debating the assigned question, including the most recent scientific data, their opinions and arguments. A meeting was held for the experts, the panel members and a large audience of intensive care physicians. The experts presented their analyses and the specific scientific data on the question for which they were responsible, and they answered the questions and comments of the panel and the public. After the public meeting, the panel met privately to draft the text answering the questions. Recommendations were formulated according to the GRADE methodology. The proposed recommendations were presented and discussed individually. The aim was not necessarily to obtain a convergent opinion of the panel members for all the proposals but rather to uncover points of agreement and points of disagreement or indecision. Each recommendation was then assessed by each panel member and scored individually from 1 (totally disagree) to 9 (strongly agree). The panel score was defined using a GRADE grid [8]. To achieve a strong recommendation, at least 70% of the participants had to agree. If there was no strong agreement, recommendations were reworded and then rescored to achieve consensus. The final text contains the conclusions and recommendations of the conference.

## Section 1: definitions, scores, oxygen therapy techniques and devices

### 1: Definitions of acute respiratory failure and respiratory distress

Acute respiratory failure (ARF) is defined as the sudden inability of the respiratory system to ensure satisfactory hematosis. A distinction is made between type I (hypoxemic without hypercapnia) and type II ARF (hypercapnic acidosis). Mixed ARF is defined as the combination of hypoxemia and hypercapnia.

In this definition, hypoxemia is characterized by PaO_2_ < 60 mmHg on ambient air, SpO_2_ < 90% on ambient air, or the need to administer oxygen to achieve PaO_2_ ≥ 60 mmHg or SpO_2_ ≥ 90%. Oxygen therapy should not be discontinued to certify the presence of hypoxemia. Hypercapnic acidosis is characterized by pH ≤ 7.35, with PaCO_2_ > 45 mmHg.

Respiratory distress is clinically defined by the combination of symptoms described in Table 1. Signs of respiratory distress may precede hypoxemic ARF, with blood gas showing an initially normal PaO_2_ and hypocapnia due to secondary hyperventilation.

**Table 1:** Clinical signs characterizing respiratory distress

Signs of impaired hematosis	Cyanosis of the extremities Headache, *flapping tremor (asterixis)*, sweating, cutaneous vasodilation, arterial hypertension, drowsiness, confusion, disorientation, consciousness disorders, coma Cardiovascular failure (tachycardia, arterial hypotension)
Signs of struggle	Polypnea or tachypnea (respiratory rate > 25 cycles per minute) Pulling with engagement of the accessory respiratory muscles (sternocleidomastoid, scalene), inspiratory supra-sternal, supra-clavicular or intercostal depression, active abdominal contraction Inability to speak
Signs of exhaustion	Bradypnea (respiratory rate < 12 cycles per minute) Paradoxical abdominal breathing (recoil of the anterior abdominal wall on inspiration)

Hyperoxemia corresponds to an increase in PaO_2_ greater than that obtained by breathing ambient air. PaO_2_ > 100–120 mmHg is usually used, in the absence of a consensus threshold.

### 2. Oxygenation scores and indices

Several oxygenation indices can be used to assess the severity of the deterioration in the patient's hematosis by associating PaO_2_ or SpO_2_ to the FiO_2_ required to obtain it, and possibly with the patient's work of breathing.

The PaO_2_/FiO_2_ ratio is used to define the severity of hypoxemia in acute respiratory distress syndrome (ARDS), characterized by a PaO_2_/FiO_2_ < 300 mmHg [9, 10]. However, this ratio requires arterial blood gas and precise measurement of Fi O_2_.

The FiO_2_ of patients treated with standard mask oxygen therapy can be estimated using the Coudroy formula [11], where Q{ _O2_} is the oxygen flow rate in L/min:

$$F_{i} O_{2} \left( \% \right) = 21 + Q_{{{\text{O}}_{2} { }}} \left( {L/min} \right) \times 3$$

To avoid arterial blood gas sampling, the SpO_2_ /FiO_2_ ratio can be used as a substitute for the Pa O_2_ /FiO_2_ ratio. SpO_2_/FiO_2_ ratios of 235 and 315 correlate with PaO_2_ /FiO_2_ ratios of 200 and 300 mmHg respectively [12]. SpO_2_ may also be over or underestimated depending on ethnicity, the patient's clinical condition and the reliability of the measurement system [13–15]. Interpretation of the SpO_2_/FiO_2_ ratio requires titration of FiO_2_ as SpO_2_ is limited to 100%.

The ROX index is defined as the ratio of SpO_2_ /FiO_2_ divided by the respiratory rate: $ROX = \frac{S_pO_2/ F_iO_2}{FR}$. In patients treated with high-flow nasal oxygen therapy (HFNC), the ROX index has been shown to be useful in predicting the success of this technique [16]. ROX index < 2.85 at H2, < 3.5 at H6 or < 4.88 at H12 of high flow nasal oxygen therapy is predictive of failure. However, the ROX index does not appear to be as effective in immunocompromised patients [17], and has not been sufficiently studied in patients with ARF in pre-hospital or emergency care. As with the Sp O_2_ /FiO_2_ ratio, interpretation of the ROX is subject to FiO_2_ titration.

### 3. Oxygen therapy techniques and devices

The aim of oxygen therapy is to re-establish sufficient hematosis to ensure tissue oxygenation. Its main indication is hypoxemic ARF. Although simple to use and generally without adverse effects, oxygen is a drug, the quantity and method of administration of which must be prescribed according to the pathology and severity of the patient. Recent experimental and clinical studies have highlighted the deleterious pulmonary, cardiovascular, neurological and metabolic effects of hyperoxemia [18–21]. In addition, in certain patients (chronic obstructive pulmonary disease (COPD), other chronic respiratory insufficiencies, morbid obesity), excessive oxygen administration can lead to or worsen hypercapnia [22, 23].

Standard oxygen therapy can be administered via different interfaces. The FiO_2_ delivered depends on the minute ventilation and the seal if a mask is used (Table 2).

**Table 2:** Flow rates and resulting FiO_2_ of conventional oxygen therapy devices

Interface	Nasal cannula	Simple mask	High concentration mask
Flow	0.5–5 L/min	5 à 8 L/min	8 à 15 L/min
FiO_2_	24–40%	40–60%	40–90%

High-flow nasal cannula oxygen therapy (HFNC) is used to deliver a humidified and heated gas mixture (air/oxygen) with flow rates ranging from 10 to 70 L/min and FiO_2_ of 21–100%.

Whether or not combined with oxygen therapy, it is possible to administer continuous positive *airway pressure (*CPAP) or tele-expiratory pressure (spontaneous ventilation with positive expiratory pressure) using several interfaces: Boussignac valve, ventilator with dedicated mode.

Ventilation consists in administration of differential pressure to the airways in order to assist, partially or completely, the work of breathing. It may be invasive or non-invasive, administered by means of an external interface (mainly a face mask). As it is not limited to the administration of oxygen, it is not included in the oxygen therapy modalities *stricto *sensu in this consensus conference.

## Section 2: Indications for oxygen therapy, targets and monitoring methods

### Question 1: What are the criteria for initiating oxygen therapy in patients with acute hypoxemic respiratory failure?

**Recommendation 1A**

**The panel suggests initiating oxygen therapy in the event of acute hypoxemic respiratory failure (panel opinion, strong agreement).**

**Recommendation 1 B**

**The panel makes no recommendation on the initiation of oxygen therapy in patients with respiratory distress without hypoxemia (insufficient quality of evidence, strong agreement).**

**Arguments**

The panel underlines the importance of initiating oxygen therapy in hypoxemic ARF. However, there is no formal threshold for hypoxemia, and the administration of oxygen has not been compared with the absence of oxygen administration during hypoxemic ARF in a controlled trial with a high level of evidence [24, 25] justifying the absence of a strong recommendation.

There is only indirect evidence of oxygen administration in hypoxemic ARF. The effect of oxygen on the symptoms of hypoxemia has been known since the nineteenth century [26]. Numerous observational studies have shown that saturation < 91% is associated with an increased risk of mortality [27, 28]. More recently, a study showed the deleterious effect of a restrictive strategy (SaO_2_ 88–92%) in ARDS [29].

There are no published data supporting the use of oxygen to reduce signs of respiratory distress in non-hypoxemic patients. A meta-analysis by Hasegawa et al. included 39 randomized controlled trials, mainly on chronic respiratory failure and palliative care patients [30] and showed no benefit of oxygen in reducing symptoms of dyspnea. A meta-analysis including COPD patients with dyspnea, mainly with SpO_2_ > 90%, showed the same lack of effect [31]. A randomized controlled trial in end-of-life patients without hypoxemia found no improvement in symptoms of respiratory distress [32]. Only one small study (28 subjects) showed a positive effect of oxygen therapy on the reduction of dyspnea in patients with moderate hypoxemia [33].

### Question 2: In patients with acute hypoxemic respiratory failure, what are the targets of oxygen saturation?

**Recommendation 2 A**

**Oxygen flow or FiO**_**2**_ **should probably be adjusted according to pulse oximetry values, to achieve:**

- **SpO**_**2**_ **ranging from 94 to 98% for patients with no risk of oxygen-induced hypercapnia (GRADE 2 + , moderate quality of evidence, low agreement).**
- **SpO**_**2**_ **ranging from 88 to 92% for patients at risk of oxygen-induced hypercapnia (GRADE 2 + , moderate quality of evidence, strong agreement).**

**Recommendation 2 B**

**The panel makes no recommendation on the “liberal” or “restrictive” strategy of oxygen therapy to be adopted during acute hypoxemic respiratory failure (insufficient quality of evidence, strong agreement).**

**Arguments**

The deleterious effects of hypoxemia and hyperoxemia have been described previously. Retrospective studies on large databases have confirmed mortality increase for both low and high oxygenation levels, describing a "U" or "J" curve, with lower mortality for target range saturation of 94 and 98%, or for PaO_2_ between 100 and 120 mmHg [34, 35]. Similar results were found in the specific context of post-cardiac arrest [36].

Large retrospective cohort studies have shown a correlation between depth of hypoxemia and mortality [37, 38]. In these studies, no association was found between hyperoxemia and mortality. Similarly, Madotto et al. showed no relationship between the occurrence of hyperoxemia and prognosis in an ancillary study of the LUNG-SAFE study [39]. In a large retrospective cohort, however, Palmer et al. showed an association between the occurrence of hyperoxemia episodes and ICU mortality, albeit with no dose–response relationship [40]. Few studies have compared the effect of different targets of SpO_2_ range. In particular, no study has evaluated the effect of low PaO_2_ values outside post-cardiac arrest [36]. The available studies have included different populations, with varying oxygenation targets. In a randomized trial, Girardis et al. showed lower ICU mortality in a "conservative" group defined as PaO_2_ between 70 and 100 mmHg or SpO_2_ between 94 and 98%, to a "conventional" group defined as PaO_2_ above 150 mmHg or SpO_2_ between 97 and 100% [41]. Many subsequent trials were negative for mortality [42–44] or organ dysfunction [45], with very heterogeneous targets per group. For several studies, the highest or "liberal" level was in fact relatively low and could be described as "restrictive" in others. Barrot et al. [29] compared the oxygenation strategy among patients ventilated for ARDS with a restrictive (PaO_2_ between 55 and 60 mmHg) and a liberal (90–105 mmHg) group, showing a significant increase in the proportion of mesenteric ischemia in the restrictive group and a low probability of a significant difference on mortality, justifying early termination of the study.

In a prospective randomized pre-hospital trial, Austin et al. showed that a titrated oxygen treatment aimed at obtaining SpO_2_ between 88 and 92% reduced mortality, hypercapnia and respiratory acidosis compared with high-flow nasal cannula oxygen therapy in acute exacerbations of chronic obstructive pulmonary disease.

### Question 3: In patients with acute hypoxemic respiratory failure, what is the role of blood gas analysis?

**Recommendation 3A**

**Patients with acute hypoxemic respiratory failure should probably not be routinely monitored by blood gas analysis (GRADE 2-, moderate quality of evidence, strong agreement).**

**Arguments**

There is no strong evidence regarding the superiority of a systematic arterial blood gas analysis strategy concerning mortality and intubation rates. However, several studies [46, 47] have shown that no systematic blood gas analysis reduces the number of samples. In one of them, there was a reduction in the duration of mechanical ventilation and length of ICU stay when gas measurements were not carried out systematically, but guided by clinical assessment [46].

Furthermore, although the incidence of complications from arterial puncture remains low [48], the severity of some (embolism, thrombosis, aneurysm, arteriovenous fistula), and the invasive and painful aspect of the procedure, particularly when repeated [49], justify limitation of its systematic prescription.

**Recommendation 3B**

**The panel suggests using venous blood gas analysis to rule out hypercapnia for the evaluation and monitoring of acute hypoxemic respiratory failure (panel opinion, strong agreement).**

**Recommendation 3 C**

**The panel suggests that arterial blood gas analysis should be performed when there is a doubt about the reliability of SpO**_**2**_**, when it is not measurable, or when PvCO**_**2**_ **is elevated, so as to confirm and quantify hypercapnia (panel opinion, strong agreement).**

**Recommendation 3 D**

**The panel suggests that arterial blood gas analysis should be performed in cases of pathological hemoglobin, suspicion or presence of methemoglobin or CO intoxication, or when there is a non-respiratory indication for arterial blood gas analysis (panel opinion, strong agreement).**

**Arguments**

The correlation between PaCO_2_ and PvCO_2_ and between arterial and venous pH seems sufficient to avoid arterial blood gas analysis [50]. A PvC O_2_ threshold below 45 mmHg almost certainly rules out arterial hypercapnia. Furthermore, a multicenter randomized study in four French emergency departments demonstrated reduced patient pain levels when venous blood gas was used instead of arterial blood gas, with no change in the clinical value of the sample [51].

However, oxygen therapy monitoring should take into consideration the biases and limitations of pulse oximetry. Aside from some studies on patient groups, most technical validation studies of pulse oximeters [52] and their certification standards have been conducted on healthy volunteers, with some confounding factors, particularly signal noise. SpO_2_ accuracy is reduced in peripheral perfusion disorders, pathological hemoglobin (methemoglobin, carboxyhemoglobin, sickle cell disease), and may pose interpretation problems in cases of skin pigmentation [53].

Moreover, there are differences in the levels obtained from one oximeter to another, with some devices overestimating and others underestimating SpO_2_ compared to SaO_2_ In a study conducted in ICU patients, the average difference between SaO_2_ and SpO_2_ approximated 4.4% [54].

### Question 4: In patients with acute hypoxemic respiratory failure, when should an arterial catheter be inserted?

**Recommendation 4**

**The panel suggests invasive blood gas monitoring in patients for whom repeated arterial sampling is indicated (panel opinion, strong agreement).**

**Arguments**

This position is justified by the painful nature of arterial blood gas sampling, particularly when repeated (see argument in Question 3).

## Section 3: Choice of oxygen therapy modality

### Question 5: Should standard oxygen therapy, high-flow nasal cannula oxygen therapy (HFNC) or continuous positive airway pressure (CPAP) be preferred in patients with acute hypoxemic respiratory failure?

**Recommendation 5 A**

**The panel makes no recommendation concerning the use of CPAP rather than standard oxygen therapy in patients with acute hypoxemic respiratory failure (insufficient quality of evidence, strong agreement).**

**Recommendation 5B**

**HFNC should probably be used rather than standard oxygen therapy in patients with hypoxemic ARF, with an oxygen flow rate > 6L/min to achieve SpO**_**2**_ > **92% or a** **PaO**_**2**_**/Fi O**_**2**_ **ratio < 200 (GRADE 2 + , moderate quality of evidence, strong agreement).**

**Arguments**

Three studies in non-COVID-19 patients and two studies in COVID-19 patients compared CPAP with conventional oxygen therapy. In non-COVID-19 patients, only one small study in hematology patients found that those treated with CPAP were less likely to be intubated [55]. The other two studies found no effect on intubation, whereas Delclaux et al. used high-flow CPAP (100 L/min oxygen) [56] and Brambilla et al. used CPAP with a Helmet interface (*Helmet CPAP*) [57]. The two trials carried out in COVID-19 patients showed contradictory results on intubation [58, 59]. However, in these trials the authors reported frequent discomfort during CPAP, leading to its being discontinued in 15 to 20% of patients. Except in hematology patients [55] no study has shown a beneficial effect of CPAP on mortality. Of note, no study has compared CPAP with HFNC. A meta-analysis including the three randomized trials comparing HFNC and conventional oxygen therapy in patients with de novo hypoxemic ARF, excluding patients with exacerbation of chronic obstructive pulmonary disease and/or hydrostatic pulmonary edema, showed that patients treated with HFNC were less likely to be intubated (Appendix 3). Nevertheless, none of the three separately analyzed randomized trials showed a significant reduction of intubation [60–62]. Frat et al. [60] found that patients treated with HFNC, compared to patients treated with conventional oxygen therapy, had a lower mortality rate, even though this was not confirmed in the two other randomized trials. The meta-analysis showed a trend towards lower mortality in patients treated with HNFC (Appendix 4).

In COVID-19 patients, two multicenter randomized trials showed that patients treated with HFNC were less likely to be intubated than those receiving conventional oxygen therapy [63, 64]. However, four other randomized trials did not confirm a beneficial effect of HFNC on intubation [58, 59, 65, 66]. This discrepancy may be explained by the severity of illness, which differs between trials, lack of power and crossovers in some trials. Nevertheless, a meta-analysis including all these studies found a beneficial effect of HFNC on intubation compared with conventional oxygen therapy (Appendix 3). In contrast, none of these randomized trials found a beneficial effect of HFNC on mortality compared with conventional oxygen therapy, even though the meta-analysis showed a trend towards lower mortality in patients treated with HNFC (Appendix 4).

Given the PaO_2_ /FiO_2_ ratio at baseline in the different trials in COVID-19 and non-COVID-19 patients and decreased intubation in patients treated with HFNC in a *post-hoc* analysis of the FLORALI study [60] in the subgroup of patients with a PaO_2_/FiO_2_ ratio < 200, the panel retained a threshold value of PaO_2_/FiO_2_ ratio < 200 or an oxygen flow rate > 6 L/min to obtain SpO_2_ > 92% as a criterion for initiation of HNFC in patients with de novo hypoxemic ARF.

## Section 4: Indications for non-invasive and invasive mechanical ventilation

### Question 6: What are the indications for non-invasive ventilation (NIV) in patients with acute hypoxemic respiratory failure?

**Recommendation 6 A**

**In the absence of intubation criteria, high-flow nasal cannula oxygen therapy (HFNC) should probably be used rather than NIV in patients with de novo acute hypoxemic respiratory failure (GRADE 2 + , moderate quality of evidence, strong agreement).**

**Arguments**

Several randomized studies have compared NIV with HFNC [60, 67–70]. None have demonstrated the superiority of any technique concerning intubation, except for Grieco et al., who found that patients treated with NIV using the Helmet device were less likely to be intubated. Frat et al. and Nair et al. found that patients treated with HFNC also tended to be intubated less frequently. A meta-analysis including all these studies showed that patients treated with HFNC tended to be less likely to be intubated (Appendix 5). The FLORALI study [60] also found a higher mortality rate in patients with de novo hypoxemic ARF treated with NIV than in patients treated with HFNC. The authors suggested that this increased mortality in patients treated with NIV might be related to increased incidence of ventilatory-induced lung injury (VILI) due to high tidal volume in patients treated with NIV. Nair et al. likewise found a trend towards higher mortality in patients treated with NIV. A meta-analysis including all these studies found a trend towards lower mortality in favor of HFNC (Appendix 6). Finally, HFNC appears to be easier to use and better tolerated than NIV.

**Recommendation 6 B**

**The panel makes no recommendation concerning NIV** ***versus*** **standard oxygen therapy in patients with de novo hypoxemic ARF, including immunocompromised patients (insufficient quality of evidence, strong agreement).**

**Arguments**

Numerous studies have compared NIV with standard oxygen therapy in patients with de novo hypoxemic ARF and have shown conflicting results on intubation. All the older studies showed that patients treated with NIV were less likely to be intubated (Appendix 5). Nevertheless, these studies had small sample size and included heterogeneous patients, 30–75% of whom suffered from acute exacerbation of chronic obstructive pulmonary disease and/or hydrostatic pulmonary edema. The two randomized multicenter trials excluding patients with acute exacerbation of chronic obstructive pulmonary disease and/or hydrostatic pulmonary edema did not find any difference between NIV and conventional oxygen therapy concerning intubation [60, 71].

In immunocompromised patients, four randomized trials compared NIV with standard oxygen therapy and/or HFNC [69, 72–74]. Only the two oldest studies showed that patients treated with NIV were less likely to be intubated, while the two most recent studies showed no superiority of NIV. This discrepancy may be explained by the fact that in Antonelli et al. and Hilbert et al., patients in the control group were treated with standard oxygen therapy alone, whereas in the control group, 40% of patients in the study by Lemiale et al. and all patients in the study by Coudroy et al. were treated with HFNC. Nevertheless, a meta-analysis including all of these studies found that patients treated with NIV were less likely to be intubated (Appendix 7). Only one study showed decreased mortality in patients treated with NIV [73]. However, a meta-analysis including all these studies showed a trend towards lower mortality with NIV (Appendix 8).

### Question 7: What are the indications for invasive mechanical ventilation in patients with acute hypoxemic respiratory failure?

**Recommendation 7 A**

**The panel suggests that in acute hypoxemic respiratory failure, the presence of any of the following criteria requires intubation (panel opinion, strong agreement):**

- **Cardiac or respiratory arrest due to hypoxemia**
- **Persistent hypoxemia despite maximal oxygenation strategy, with PaO**_**2**_**/FiO**_**2**_ < **60 mm Hg and/or SpO**_**2**_ <** 88%.**

**Recommendation 7 B**

**The panel suggests that in acute hypoxemic respiratory failure, the presence of one or more of the following criteria should lead to consideration of intubation (panel opinion, strong agreement):**

- **Shock requiring vasopressor**
- **Clinical signs of respiratory distress**
- **Appearance or worsening of vigilance disorders**
- **Worsening hypoxemia despite maximal oxygenation strategy**
- **Persistent hypoxemia despite maximal oxygenation strategy, with Pa O**_**2**_**/FiO**_**2**_ < **100 mm Hg or SpO**_**2**_ <** 92%.**
- **Respiratory or mixed acidosis with pH < 7.30**
- **Tachypnea with respiratory rate > 30 or worsening respiratory rate**
- **Bronchial congestion or copious secretions**
- **Recurrent desaturation episodes with SpO**_**2**_ < **86%**
- **Agitation**
- **Intolerance to oxygenation modality**

**Arguments**

No study has compared presence vs. absence of intubation in acute hypoxemic respiratory failure. The present recommendation was therefore drafted following a vote by the experts consulted, using the DELPHI method.

Numerous studies comparing different oxygen therapy or non-invasive ventilation strategies have proposed need for invasive mechanical ventilation as an endpoint. The risks associated with intubation are manifold, including procedural failure, ventilator-associated pneumonia [75], induced lung injury, hemodynamic effects and respiratory muscle amyotrophy [76]. However, delayed intubation has been identified as a risk factor for mortality in several studies [77] although severity at the time of the intubation decision may be a confounding factor.

Identified failure of an oxygenation strategy and/or failure of non-invasive ventilation and the decision to switch to invasive ventilation consequently aim to avoid exposing the patient to unnecessary intubation.

Hypoxemic cardiac arrest and failure of a maximal oxygenation strategy with persistent deep hypoxemia are the two situations identified by the panel as systematically requiring intubation. Aside from these situations, the decision to intubate must be individualized, taking into account the patient's oxygenation status, progress, tolerance of the strategy initiated, and any other organ failure.

## Section 5: The role of adjuvant therapies: awake prone position and physiotherapy.

### Question 8: In patients with acute hypoxemic respiratory failure, should awake prone position be used?

**Recommendation 8A**

**The panel makes no recommendation concerning awake prone position in acute hypoxemic respiratory failure not related to COVID-19 (insufficient quality of evidence, strong agreement).**

**Recommendation 8 B**

**In acute hypoxemic respiratory failure related to COVID-19, awake prone position should probably be used to reduce the need for intubation in patients requiring high-flow oxygen therapy (GRADE 2 + , moderate quality of evidence, strong agreement).**

**Arguments**

The clinical data concerning awake prone position (APP) are recent. All relevant studies have been conducted in patients with COVID-19-related type I ARF. Out of the prospective studies, only 9 were multicenter; 8 were randomized [78–85], and the other was non-randomized [86].

However, these studies did not show any significant reduction in mortality or length of stay and were discordant concerning intubation. Moreover, while the meta-analytical data available to date suggest no benefit of APP on patient survival or length of stay, they confirm a reduced rate of intubation [87–91].

The largest study was an international meta-trial, *i.e.* a simultaneous multicenter prospective analysis of individual patient data from six nationwide randomized trials, and found a beneficial effect on the intubation rate [80]. The patients received high-flow oxygen therapy. Without evidence favoring other oxygenation modalities, it therefore seems legitimate to recommend high-flow oxygen therapy when using APP.

APP should remain an adjuvant method and should not delay intubation. It should not be considered as a rescue method.

Pending the results of specific studies to come, the data on APP cannot be extrapolated to hypoxemic ARF in non-COVID 19 patients, [92].

To date, there are no data concerning the time elapsed between symptom onset and APP initiation. In the largest positive APP cohort, it was only performed in ICUs [84]. Aside from exceptional health conditions, given the high risk of progression to intubation during APP (33% in the study by Ehrmann et al.) and the increased workload for nursing teams, APP should be used only in ICUs.

It seems difficult, due to the substantial heterogeneity of studies, to make precise recommendations concerning APP session duration or minimum daily duration. However, Ehrmann et al. suggested that longer sessions were associated with greater benefit in terms of risk of intubation or death [80]. Median APP duration was 5.0 h/d [1.6–8.8]. Ibarra-Estrada et al. found a lower rate of intubation or death in patients having received sessions lasting more than 8 h [84].

The rate of adverse events associated with APP seems low [80]. On the other hand, patient discomfort and/or inadequate compliance are the main elements of concern with regard to APP tolerance. The pathophysiology of APP effects has only occasionally been explored. Some studies seem to show that inspiratory pressures increased in association with increased airway resistance [93]. APP should therefore probably be avoided in the most polypneic patients, so as not to increase the risk of self-inflicted lung injury.

### Question 9: In patients with acute hypoxemic respiratory failure, what is the role of physiotherapy?

**Recommendation 9 A**

**The panel suggests physiotherapy to promote lung recruitment in clinically stable patients with acute hypoxemic respiratory failure requiring ICU admission (panel opinion, strong agreement).**

**Arguments**

All physiotherapy treatments must be the result of a team discussion taking into consideration the patient's level of organ failure and the underlying disease [94]. To date, no randomized controlled trial has evaluated the positive contribution of physiotherapy in patients undergoing oxygen therapy for ARF. Physiotherapy, whether motor or respiratory, cannot be considered as a reference treatment.

Two studies have shown that motor physiotherapy (bed exercises, chair positioning, cycloergometer) improves ventilation distribution in the posterior lung regions [95, 96]. In addition, Hickmann et al. found improved hematosis, with an increased PaO_2_/FiO_2_ ratio, especially in the most severe patients [96]. However, these effects were not sustained over time and regressed at the end of the procedure. In addition to respiratory function, motor physiotherapy has other objectives: prevention of bedsores or sarcopenia, maintenance of joint amplitudes, improved comfort…[97].

There are currently no studies on respiratory physiotherapy in de novo ARF patients. While the recommendations of the American Association for Respiratory Care and the British Thoracic Society address the overall role of respiratory physiotherapy, they do not specifically consider resuscitation or intensive care patients with ARF [98, 99]. In these recommendations, respiratory physiotherapy techniques are reserved for ARF patients with additional bronchial congestion. The panel members suggested that the state of congestion of an ARF patient, and the indication for respiratory physiotherapy be assessed by the physiotherapist.

**Recommendation 9 B**

**The panel makes no recommendation concerning systematic respiratory physiotherapy in acute hypoxemic respiratory failure (insufficient quality of evidence, strong agreement).**

There are currently no studies on respiratory physiotherapy in ARF patients. While the recommendations of the American Association for Respiratory Care and the British Thoracic Society address the overall role of respiratory physiotherapy, they do not specifically consider ICU patients [98, 99]. In these recommendations, respiratory physiotherapy techniques are reserved for ARF patients with additional bronchial congestion. The panel suggests that congestion and the indication for respiratory physiotherapy be assessed by the physiotherapist.

## Section 6: organizational measures

### Question 10: In patients with acute hypoxemic respiratory failure, which criteria necessarily lead to ICU admission?

**Recommendation 10 A**

**The panel suggests that patients receiving conventional oxygen therapy and showing clinical signs of respiratory distress should be managed in an ICU (panel opinion, strong agreement).**

**Recommendation 10B**

**The panel suggests that patients receiving HFNC, CPAP or NIV should be managed in an ICU (panel opinion strong agreement).**

**Arguments**

There has been no randomized trial comparing admission to the ICU vs. hospital ward for patients under oxygen therapy with clinical signs of respiratory distress. However, it seems reasonable for these patients to be admitted to an ICU to ensure appropriate care and monitoring.

When treatment with HFNC, CPAP or NIV is decided, the patient should be directed to an ICU to rapidly identify signs of poor tolerance, respiratory distress and in order to avoid delayed intubation [68]. In these patients, the rate of secondary intubation appears high, around 30 to 40%, justifying admission to critical care [100]. While initiation of these techniques can begin in an emergency ward, it should not delay transfer to the ICU.

In the specific context of exceptional health situations, use of HFNC, CPAP or NIV might be considered outside ICUs [58, 101].

## Section 7: Ethical considerations

### Question 11: Which oxygenation device should be preferred for patients for whom a do-not-intubate decision has been made?

**Recommendation 11**

***The panel makes no recommendation regarding the preferred oxygenation device for patients for whom a do-not-intubate decision has been made (insufficient quality of evidence, strong agreement).***

Patients with ARF for whom a do-not-intubate decision has been made represent about 25% of patients with ARF under HFNC or NIV [102], and therefore a common situation.

According to the panel, the probability of survival from the acute episode should be considered when deciding on the most suitable oxygenation device. In these patients, HFNC could improve comfort. A randomized controlled trial in oncology showed improved comfort-related outcomes (dyspnea score, dry mouth, sleep quality) in patients treated with HFNC for 72 h versus conventional oxygen [103].

Another RCT demonstrated an improved dyspnea score on the modified Borg scale when comparing HFNC and conventional oxygen in hypoxemic ARF patients with a “do-not-intubate” decision [104].

Retrospective studies and single-center cohorts (with small sample sizes and heterogeneous outcome measures) also converge towards positive effects on comfort, maintenance of oral intake, and a low rate of complications or poor tolerance with HFNC versus conventional oxygen therapy [105–107].

There are no data on the impact of HFNC on mortality in this population. NIV in these patients not only does not improve survival, but also seems to decrease patient comfort (nutrition, communication, tolerance) [108], 109].

## Supplementary Information

Supplementary material 1.

Supplementary material 2.

Supplementary material 3.

Supplementary material 4.

Supplementary material 5.

Supplementary material 6.

Supplementary material 7.

Supplementary material 8.

## Data Availability

Not applicable.
