# Supplementary material for: Oxygen therapy in acute hypoxemic respiratory failure: guidelines from the SRLF-SFMU consensus conference
Source: Ann Intensive Care. 2024 Sep 5;14:140. doi: 10.1186/s13613-024-01367-2 (PMC11377397; doi:10.1186/s13613-024-01367-2)
Supplement: Supplementary file 1 — Supplementary material 1. [file 13613_2024_1367_MOESM1_ESM.docx]

**Questions PICO**

**PICO 1 : critères d’indication d’une O2 thérapie**

| Population | Patient en IRA/DRA |
| --- | --- |
| Intervention | Instauration d’une oxygénothérapie sur critères cliniques (+ SpO2) **ET** GDS artériel |
| Comparaison | Instauration d’une oxygénothérapie sur critères cliniques/SpO2 uniquement |
| Outcome | Mortalité, recours à l’IOT, hématose, durée de VM, durée de séjour |

**PICO 2 : cibles d’O2 thérapie**

| Population | Patient en IRA nécessitant une O2 thérapie |
| --- | --- |
| Intervention | Oxygénothérapie objectif restrictif (seuils de SpO2 ou PaO2 à définir) |
| Comparaison | Oxygénothérapie objectif libéral (seuils de SpO2 ou PaO2 à définir) |
| Outcome | Mortalité, recours à l’IOT, hématose, durée de VM, durée de séjour |

**PICO 3 : Surveillance systématique des GDS**

| Population | Patient en IRA nécessitant une O2 thérapie |
| --- | --- |
| Intervention | Surveillance systématique des GDS artériels et ou veineux |
| Comparaison | Pas de surveillance systématique des GDS |
| Outcome | Mortalité, recours à l’IOT, hématose, durée de VM, durée de séjour, confort du patient |

**PICO 4 : monitorage invasif vs. non invasif**

| Population | Patient en IRA nécessitant une O2 thérapie |
| --- | --- |
| Intervention | Monitorage non invasif (sans cathéter artériel) |
| Comparaison | Monitorage invasif (avec cathéter artériel) |
| Outcome | Mortalité, recours à l’IOT, hématose, durée de VM, durée de séjour, confort du patient |

**PICO 5 : comparaison des modalités d’O2 thérapie sur le pronostic**

| Population | Patients en IRA hypoxémique, non-hypercapnique sous oxygénothérapie (COVID et non COVID, inclure les immunodéprimés)  Exclus :OAP , décompensation aiguë d’une insuffisance respiratoire chronique (BPCO sévère, sous support d’oxygénation au long) |
| --- | --- |
| Intervention | - - O2 conventionnelle   - OHD   - CPAP avec/ou OHD avec/non helmet (la VNI est traitée dans un autre champ le n°4)   - OHD définir un débit minimum (40 L/MIN ?) pour parler d’OHD |
| Comparaison | - - O2 conventionnelle   - OHD   - CPAP avec/ou OHD avec/non helmet (la VNI est traitée dans un autre champ le n°4) |
| Outcome | - - Les majeurs : intubation / mortalité (J28, J90, Réa, hôpital choisir le dénominateur commun dans les études retenues)   - « escalation » ou conversion vers ventilation mécanique (VNI ou ventilation invasive)   - Les cliniques : fréquence respiratoire, confort, hématose (PaO2, P/F voire PaCO2)   - Sécurité : tolérance, contamination environnement (bio-aerosolisation)   - Devenir : durée VM (voire VFree-day 28 si possible statistiquement), duré de séjour en réa, hôpital |

**PICO 6 Indications de VNI**

| Population | Patient en IRA nécessitant une O2 thérapie |
| --- | --- |
| Intervention | Ventilation non invasive |
| Comparaison | O2 conventionnelle, OHD/ ou CPAP/ |
| Outcome | Mortalité, recours à l’intubation, hématose, durée de VM, durée de séjour, confort, complications |

**PICO 7 DV vigile**

| Population | Patient en IRA nécessitant une O2 thérapie |
| --- | --- |
| Intervention | DV vigile |
| Comparaison | Pas de DV vigile |
| Outcome | Mortalité, recours à l’IOT, hématose, durée de VM, durée de séjour, tolérance |

**PICO 8 : kinésithérapie**

| Population | Patient en IRA nécessitant une O2 thérapie |
| --- | --- |
| Intervention | Kinésithérapie (respiratoire, motrice, mobilisation, levé, fauteuil) |
| Comparaison | Pas de kinésithérapie |

**PICO 9 Orientation/ « triage » des patients en IRA**

| Population | Patient en IRA nécessitant une O2 thérapie conventionnelle, OHD et CPAP (hors conditions sanitaires exceptionnelles) |
| --- | --- |
| Intervention | Admission en soins critiques |
| Comparaison | Hospitalisation conventionnelle |
| Outcome | Mortalité, transfert en réanimation, recours à l’IOT, hématose, durée de VM, durée de séjour |

**PICO 10 O2 thérapie pour les patients chez qui une décision de non intubation a été prise**

| Population | Patient en IRA nécessitant une O2 thérapie avec décision de ne pas recourir à l’intubation |
| --- | --- |
| Intervention | OHD/CPAP/O2 conventionnelle |
| Comparaison | OHD/CPAP/O2 conventionnelle |
| Outcome | Mortalité, hématose, durée de VM, durée de séjour, confort, vécu des patients, vécu des familles, vécu des soignants |
