# Supplementary material for: Oxygen therapy in acute hypoxemic respiratory failure: guidelines from the SRLF-SFMU consensus conference
Source: Ann Intensive Care. 2024 Sep 5;14:140. doi: 10.1186/s13613-024-01367-2 (PMC11377397; doi:10.1186/s13613-024-01367-2)
Supplement: Supplementary file 2 — Supplementary material 2. [file 13613_2024_1367_MOESM2_ESM.zip › GRADE PICO 9.docx]

**Question 9 « Quels sont les critères d’admission en soins critiques pour les patients sous O2 thérapie au cours de l’IRA »**

Clé de recherche : (((Intensive care unit[MeSH Terms]) AND (Patient Admission[MeSH Terms])) AND (respiratory insufficiency[MeSH Terms])) OR (((Emergency Ward[MeSH Terms]) AND (Mortality[MeSH Terms])) AND (acute respiratory failure[MeSH Terms]))AND 2012/01/01:2023/06/16[dp]

Après exclusion des papiers sans abstract, non en langue anglaise, et portant sur des patients de pédiatrie la recherche a identifié 53 papiers dont 35 en langue anglaise, avec abstracts, portants sur les adultes.

Après sélection sur titre et abstract la recherche a identifié les 13 papiers ci-dessous (Annexe 1).

Après lecture des articles, seuls 8 étaient en lien avec la question posée et ont été analysés (Annexe 2) et références (page 2).

Les papiers sélectionnés ne répondent qu’indirectement à la question PICO. Ils soulignent l’hétérogénéité des pratiques selon les systèmes de soin (niveau de preuve faible à très faible) [1-3], la faisabilité des techniques d’oxygénation telles que la VNI ou l’optiflow, à la limite que les patients avec IRA de novo sont relativement rares dans l’étude portant sur la VNI et l’admission en réanimation après initiation la régle. Enfin, plusieurs papiers, là encore de niveau de preuve faible à très faible, démontrent le sur-risque de décés en cas d’admission retardée (avec une définition allant de plus de 2h à plus de 24h) [4-7]. Enfin le dernier papier, bien qu’évaluant la stratégie d’admission en réanimation, évalue principalement le site d’hospitalisation avant admission en réanimation sans ajuster de manière adéquate pour les principaux facteurs confondants et sans analyser les caractéristiques du patient aux urgences [8]. Cette dernière étude, observationnelle, semble présenter des biais sérieux (données recueillies à l’admission en réanimation plutôt qu’avant l’admission), les données rapportées sont peu interprétables dans le cadre de la question PICO, le manque de précision est sérieux, les potentiels facteurs confondants pourraient changer le sens de l’association, et aucun effet dose réponse n’est observé (très faible niveau de preuve) [8].

**References**

1. Marjanovic et al. Am J Emerg Med 2020
2. Ugurlu et al. Lung 2015
3. Goel et al. Respir Care 2020
4. Mokart et al. Leuk Lymph 2013
5. Hung et al. Critical Care 2014
6. Anesi et al. Annals ATS 2023
7. Hsieh et al. Am J Emerg Med 2017
8. Valentini et al. Respir Care 2013

**Cotation GRADE des papiers sélectionnés**

1. **Papiers évaluant la faisabilité de la VNI ou l’oxygéne à haut débit humidifié**

**
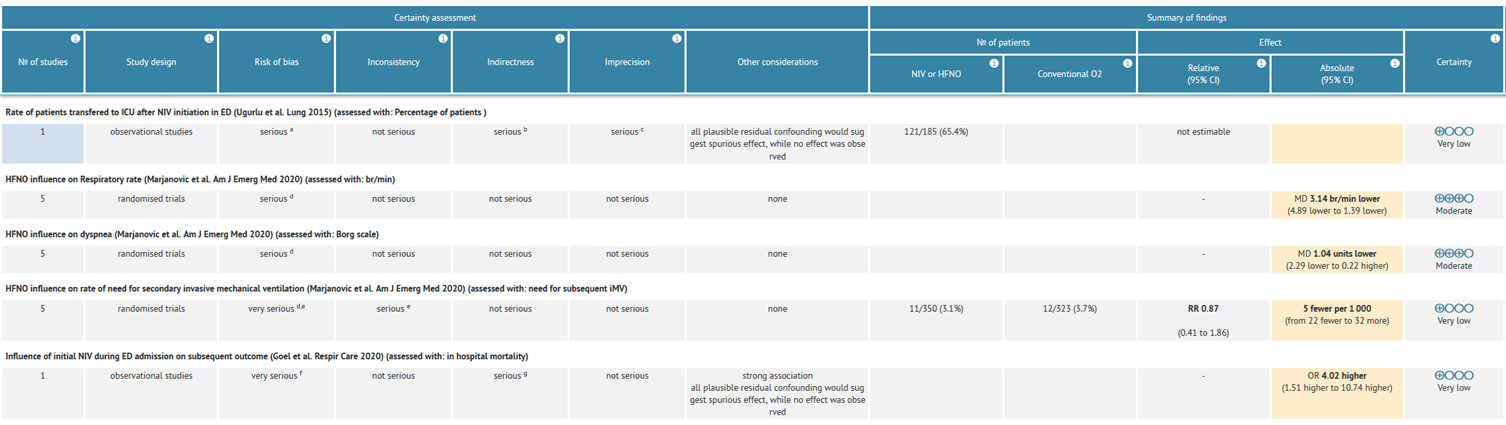
**

1. **Papiers évaluant l’influence d’un retard à l’admission en réanimation**

**
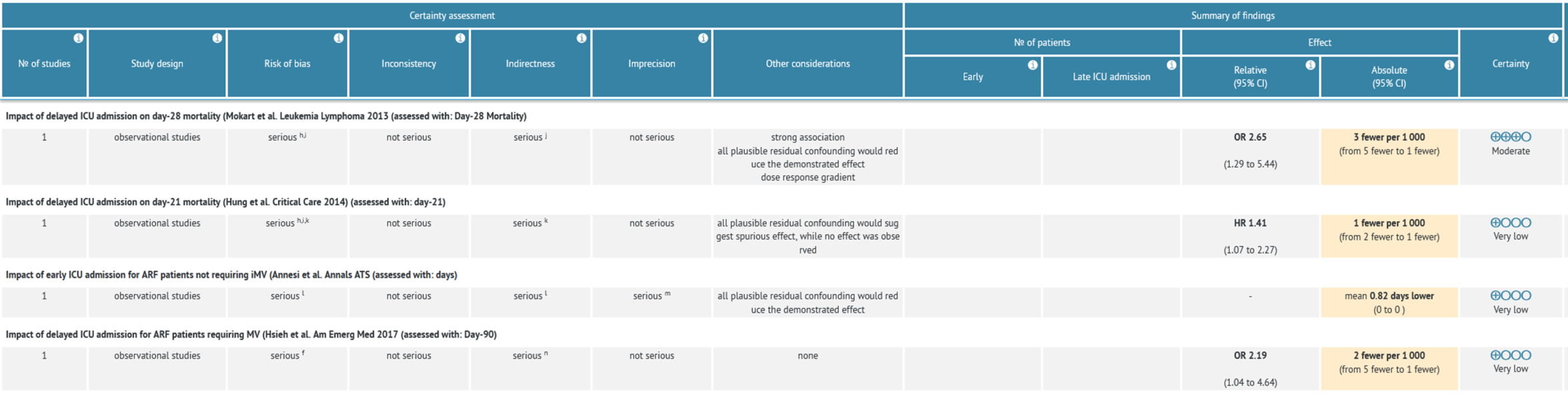
**

**Annexe 1- Articles identifiés répondants aux critéres prédéfinis et lus**

1. **PMID- 36085732**

OWN - NLM

STAT- MEDLINE

DCOM- 20220913

LR - 20221019

IS - 2694-0604 (Electronic)

IS - 2375-7477 (Linking)

VI - 2022

DP - 2022 Jul

TI - Early ICU Mortality Prediction for Respiratory Failure by A Regression-Based

Hazard Markov Model.

PG - 2651-2654

LID - 10.1109/EMBC48229.2022.9871309 [doi]

AB - Respiratory failure is one of the major causes of death in critical care units.

While respiratory failure could come with the acute symptoms progressively, an

early warning model is urgently required to assess mortality risks in advance. To

this end, an early mortality risk prediction in patients who suffer respiratory

failures can provide support for timely decision making of clinical treatment and

medical resource management. In the study, we propose a dynamic modeling approach

for early mortality risk prediction of at-risk patients with respiratory failure

based on the first 24 hours of ICU physiological data. Our proposed model is

validated on the eICU Collaborate Research Database. We achieve high AUROC

performance of around 80% and significantly improved AUCPR by 4% from Day 4 to

Day 6 since ICU admission, compared to the state-of-art prediction models.

Furthermore, we show the survival probability curve that contains the

time-varying information for early ICU admission patients.

FAU - Yin, Yilin

AU - Yin Y

FAU - Chou, Chun-An

AU - Chou CA

LA - eng

PT - Journal Article

PL - United States

TA - Annu Int Conf IEEE Eng Med Biol Soc

JT - Annual International Conference of the IEEE Engineering in Medicine and Biology

Society. IEEE Engineering in Medicine and Biology Society. Annual International

Conference

JID - 101763872

SB - IM

MH - Hospitalization

MH - Humans

MH - *Intensive Care Units

MH - Patient Admission

MH - Probability

MH - *Respiratory Insufficiency/diagnosis

EDAT- 2022/09/11 06:00

MHDA- 2022/09/14 06:00

CRDT- 2022/09/10 01:03

PHST- 2022/09/10 01:03 [entrez]

PHST- 2022/09/11 06:00 [pubmed]

PHST- 2022/09/14 06:00 [medline]

AID - 10.1109/EMBC48229.2022.9871309 [doi]

PST - ppublish

SO - Annu Int Conf IEEE Eng Med Biol Soc. 2022 Jul;2022:2651-2654. doi:

10.1109/EMBC48229.2022.9871309.

1. **PMID- 26640903**

OWN - NLM

STAT- MEDLINE

DCOM- 20160915

LR - 20170506

IS - 2146-8427 (Electronic)

IS - 1304-0855 (Linking)

VI - 13 Suppl 3

DP - 2015 Nov

TI - Late Intensive Care Unit Admission in Liver Transplant Recipients: 10-Year

Experience.

PG - 15-21

AB - OBJECTIVES: We evaluated late intensive care unit admission in liver transplant

recipients to identify incidences and causes of acute respiratory failure in the

postoperative period and to compare these results with results in patients who

did not have acute respiratory failure. MATERIALS AND METHODS: We retrospectively

screened the data of 173 consecutive adult liver transplant recipients from

January 2005 through March 2015 to identify patients with late admission (> 30 d

posttransplant) to an intensive care unit. Patients were divided into 2 groups:

patients with and without acute respiratory failure. Acute respiratory failure

was defined as severe dyspnea, respiratory distress, decreased oxygen saturation,

hypoxemia or hypercapnia on room air, or need for noninvasive or invasive

mechanical ventilation. Demographic, laboratory, clinical, and respiratory data

were collected. Model for End-Stage Liver Disease, Acute Physiology and Chronic

Health Evaluation II, and Sequential Organ Failure Assessment scores; lengths of

intensive care unit and hospital stays; and hospital mortality were assessed.

RESULTS: Among 173 patients, 37 (21.4%) were admitted to an intensive care unit,

including 22 (59.5%) with acute respiratory failure. The leading cause of acute

respiratory failure was pneumonia (n = 19, 86.4%). Patients with acute

respiratory failure had significantly lower levels of albumin before intensive

care unit admission (P = .003). In patients with acute respiratory failure,

severe sepsis and septic shock were more frequently observed and tracheotomy was

more frequently performed (P = .041). CONCLUSIONS: Acute respiratory failure

developed in 59.5% of liver transplant recipients with late intensive care unit

admission. The leading cause was pneumonia, with this group of patients having

higher requirements for invasive mechanical ventilation and tracheotomy, longer

stays in an intensive care unit, and higher mortality.

FAU - Atar, Funda

AU - Atar F

AD - From the Department of Anesthesiology and Reanimation, Başkent University Faculty

of Medicine, Ankara, Turkey.

FAU - Gedik, Ender

AU - Gedik E

FAU - Kaplan, Şerife

AU - Kaplan Ş

FAU - Zeyneloğlu, Pınar

AU - Zeyneloğlu P

FAU - Pirat, Arash

AU - Pirat A

FAU - Haberal, Mehmet

AU - Haberal M

LA - eng

PT - Journal Article

PL - Turkey

TA - Exp Clin Transplant

JT - Experimental and clinical transplantation : official journal of the Middle East

Society for Organ Transplantation

JID - 101207333

SB - IM

MH - Acute Disease

MH - Adult

MH - Female

MH - Hospital Mortality

MH - Humans

MH - Incidence

MH - *Intensive Care Units

MH - Length of Stay

MH - Liver Transplantation/*adverse effects/mortality

MH - Male

MH - Middle Aged

MH - *Patient Admission

MH - Pneumonia/diagnosis/*etiology/mortality/therapy

MH - Respiration, Artificial

MH - Respiratory Insufficiency/diagnosis/*etiology/mortality/therapy

MH - Retrospective Studies

MH - Risk Factors

MH - Time Factors

MH - Tracheostomy

MH - *Transplant Recipients

MH - Treatment Outcome

EDAT- 2015/12/08 06:00

MHDA- 2016/09/16 06:00

CRDT- 2015/12/08 06:00

PHST- 2015/12/08 06:00 [entrez]

PHST- 2015/12/08 06:00 [pubmed]

PHST- 2016/09/16 06:00 [medline]

AID - 10.6002/ect.tdtd2015.O10 [doi]

PST - ppublish

SO - Exp Clin Transplant. 2015 Nov;13 Suppl 3:15-21. doi: 10.6002/ect.tdtd2015.O10.

1. **PMID- 28108054**

OWN - NLM

STAT- MEDLINE

DCOM- 20171116

LR - 20181113

IS - 0736-4679 (Print)

IS - 0736-4679 (Linking)

VI - 52

IP - 4

DP - 2017 Apr

TI - Acute Respiratory Compromise in the Emergency Department: A Description and

Analysis of 3571 Events from the Get With the Guidelines-Resuscitation(®)

Registry.

PG - 393-402

LID - S0736-4679(16)31115-5 [pii]

LID - 10.1016/j.jemermed.2016.11.060 [doi]

AB - BACKGROUND: Respiratory events requiring the use of assisted ventilation are

relatively common in the emergency department (ED), and can be associated with

substantial morbidity and mortality. OBJECTIVE: The aim of this study was to

describe and elucidate patient and event characteristics associated with

mortality and progression to cardiac arrest in ED patients with acute respiratory

compromise. METHODS: Data were obtained from the multicenter Get With the

Guidelines-Resuscitation® registry. We included patients with acute respiratory

compromise defined as absent, agonal, or inadequate respiration that required

emergency assisted ventilation. All adult patients between January 2005 and

December 2014 with an index event in the ED were included. We used multivariable

logistic regression models to assess the association between patient and event

characteristics and in-hospital mortality, with cardiac arrest during the event

as a secondary outcome. RESULTS: A total of 3571 events were included. The

in-hospital mortality was 34%. Twelve percent of events progressed to cardiac

arrest, with a subsequent 82% in-hospital mortality. When adjusting for patient

and event characteristics, we found no temporal changes in in-hospital mortality

from 2005 to 2014. Several characteristics were associated with increased

mortality, such as pre-event hypotension, septicemia, and acute stroke.

Similarly, multiple characteristics, including pre-event hypotension, were

associated with progression to cardiac arrest. CONCLUSIONS: Patient with acute

respiratory compromise in the ED had an in-hospital mortality of 34% in the

current study. These patients also have a high risk of progressing to cardiac

arrest, with a subsequent increase in in-hospital mortality to 82%. Potentially

reversible characteristics, such as hypotension before the event, showed a strong

association to in-hospital mortality, along with multiple other patient and event

characteristics.

CI - Copyright © 2016 Elsevier Inc. All rights reserved.

FAU - Karlsson, Carl Mathias

AU - Karlsson CM

AD - Department of Emergency Medicine, Beth Israel Deaconess Medical Center, Boston,

Massachusetts; Research Center for Emergency Medicine, Aarhus University

Hospital, Aarhus, Denmark.

FAU - Donnino, Michael W

AU - Donnino MW

AD - Department of Emergency Medicine, Beth Israel Deaconess Medical Center, Boston,

Massachusetts; Department of Medicine, Division of Pulmonary, Critical Care, and

Sleep Medicine, Beth Israel Deaconess Medical Center, Boston, Massachusetts.

FAU - Kirkegaard, Hans

AU - Kirkegaard H

AD - Research Center for Emergency Medicine, Aarhus University Hospital, Aarhus,

Denmark.

FAU - Cocchi, Michael N

AU - Cocchi MN

AD - Department of Emergency Medicine, Beth Israel Deaconess Medical Center, Boston,

Massachusetts; Department of Anesthesia Critical Care, Division of Critical Care,

Beth Israel Deaconess Medical Center, Massachusetts.

FAU - Chase, Maureen

AU - Chase M

AD - Department of Emergency Medicine, Beth Israel Deaconess Medical Center, Boston,

Massachusetts.

FAU - Andersen, Lars W

AU - Andersen LW

AD - Department of Emergency Medicine, Beth Israel Deaconess Medical Center, Boston,

Massachusetts; Research Center for Emergency Medicine, Aarhus University

Hospital, Aarhus, Denmark; Department of Anesthesiology, Aarhus University

Hospital, Aarhus, Denmark.

CN - American Heart Association's Get With the Guidelines-Resuscitation® Investigators

LA - eng

GR - K23 GM101463/GM/NIGMS NIH HHS/United States

GR - K24 HL127101/HL/NHLBI NIH HHS/United States

PT - Journal Article

DEP - 20170117

PL - United States

TA - J Emerg Med

JT - The Journal of emergency medicine

JID - 8412174

SB - IM

MH - Aged

MH - Aged, 80 and over

MH - *Disease Progression

MH - Emergency Service, Hospital/organization & administration

MH - Female

MH - Guideline Adherence

MH - Heart Arrest/*etiology/mortality

MH - *Hospital Mortality

MH - Humans

MH - Hypotension/mortality

MH - Male

MH - Middle Aged

MH - Multivariate Analysis

MH - Registries/statistics & numerical data

MH - Regression Analysis

MH - Respiratory Insufficiency/*etiology/mortality

MH - *Respiratory Physiological Phenomena

MH - Sepsis/mortality

MH - Stroke/mortality

MH - United States

PMC - PMC5588155

MID - NIHMS894146

OTO - NOTNLM

OT - acute respiratory compromise

OT - cardiac arrest

OT - emergency department

OT - intubation

OT - outcomes

EDAT- 2017/01/22 06:00

MHDA- 2017/11/29 06:00

CRDT- 2017/01/22 06:00

PHST- 2016/07/27 00:00 [received]

PHST- 2016/11/16 00:00 [revised]

PHST- 2016/11/30 00:00 [accepted]

PHST- 2017/01/22 06:00 [pubmed]

PHST- 2017/11/29 06:00 [medline]

PHST- 2017/01/22 06:00 [entrez]

AID - S0736-4679(16)31115-5 [pii]

AID - 10.1016/j.jemermed.2016.11.060 [doi]

PST - ppublish

SO - J Emerg Med. 2017 Apr;52(4):393-402. doi: 10.1016/j.jemermed.2016.11.060. Epub

2017 Jan 17.

1. **PMID- 25148726**

OWN - NLM

STAT- MEDLINE

DCOM- 20150916

LR - 20220318

IS - 1466-609X (Electronic)

IS - 1364-8535 (Print)

IS - 1364-8535 (Linking)

VI - 18

IP - 4

DP - 2014 Aug 23

TI - Determining delayed admission to intensive care unit for mechanically ventilated

patients in the emergency department.

PG - 485

LID - 10.1186/s13054-014-0485-1 [doi]

LID - 485

AB - INTRODUCTION: The adverse effects of delayed admission to the intensive care unit

(ICU) have been recognized in previous studies. However, the definitions of

delayed admission varies across studies. This study proposed a model to define

"delayed admission", and explored the effect of ICU-waiting time on patients'

outcome. METHODS: This retrospective cohort study included non-traumatic adult

patients on mechanical ventilation in the emergency department (ED), from July

2009 to June 2010. The primary outcomes measures were 21-ventilator-day mortality

and prolonged hospital stays (over 30 days). Models of Cox regression and

logistic regression were used for multivariate analysis. The non-delayed

ICU-waiting was defined as a period in which the time effect on mortality was not

statistically significant in a Cox regression model. To identify a suitable

cut-off point between "delayed" and "non-delayed", subsets from the overall data

were made based on ICU-waiting time and the hazard ratio of ICU-waiting hour in

each subset was iteratively calculated. The cut-off time was then used to

evaluate the impact of delayed ICU admission on mortality and prolonged length of

hospital stay. RESULTS: The final analysis included 1,242 patients. The time

effect on mortality emerged after 4 hours, thus we deduced ICU-waiting time in

ED > 4 hours as delayed. By logistic regression analysis, delayed ICU admission

affected the outcomes of 21 ventilator-days mortality and prolonged hospital

stay, with odds ratio of 1.41 (95% confidence interval, 1.05 to 1.89) and 1.56

(95% confidence interval, 1.07 to 2.27) respectively. CONCLUSIONS: For patients

on mechanical ventilation at the ED, delayed ICU admission is associated with

higher probability of mortality and additional resource expenditure. A benchmark

waiting time of no more than 4 hours for ICU admission is recommended.

FAU - Hung, Shih-Chiang

AU - Hung SC

FAU - Kung, Chia-Te

AU - Kung CT

FAU - Hung, Chih-Wei

AU - Hung CW

FAU - Liu, Ber-Ming

AU - Liu BM

FAU - Liu, Jien-Wei

AU - Liu JW

FAU - Chew, Ghee

AU - Chew G

FAU - Chuang, Hung-Yi

AU - Chuang HY

FAU - Lee, Wen-Huei

AU - Lee WH

FAU - Lee, Tzu-Chi

AU - Lee TC

LA - eng

PT - Journal Article

DEP - 20140823

PL - England

TA - Crit Care

JT - Critical care (London, England)

JID - 9801902

SB - IM

MH - APACHE

MH - Aged

MH - Confidence Intervals

MH - Critical Care

MH - Diagnosis-Related Groups

MH - Emergency Service, Hospital/*organization & administration/statistics & numerical

data

MH - Female

MH - Glasgow Coma Scale

MH - Hospital Bed Capacity

MH - *Hospital Mortality

MH - Humans

MH - Intensive Care Units/economics/organization & administration/*statistics &

numerical data

MH - Length of Stay/economics/*statistics & numerical data

MH - Logistic Models

MH - Male

MH - Odds Ratio

MH - Patient Admission/economics/*statistics & numerical data

MH - Proportional Hazards Models

MH - Respiration, Artificial/*mortality/standards

MH - Respiratory Insufficiency/etiology/*mortality/therapy

MH - Retrospective Studies

MH - Taiwan/epidemiology

MH - Time Factors

MH - Triage/organization & administration/standards

PMC - PMC4175615

EDAT- 2014/08/26 06:00

MHDA- 2015/09/17 06:00

CRDT- 2014/08/24 06:00

PHST- 2014/03/29 00:00 [received]

PHST- 2014/07/29 00:00 [accepted]

PHST- 2014/08/24 06:00 [entrez]

PHST- 2014/08/26 06:00 [pubmed]

PHST- 2015/09/17 06:00 [medline]

AID - s13054-014-0485-1 [pii]

AID - 485 [pii]

AID - 10.1186/s13054-014-0485-1 [doi]

PST - epublish

SO - Crit Care. 2014 Aug 23;18(4):485. doi: 10.1186/s13054-014-0485-1.

1. **PMID- 27742520**

OWN - NLM

STAT- MEDLINE

DCOM- 20170320

LR - 20230725

IS - 1532-8171 (Electronic)

IS - 0735-6757 (Linking)

VI - 35

IP - 1

DP - 2017 Jan

TI - Impact of delayed admission to intensive care units on patients with acute

respiratory failure.

PG - 39-44

LID - S0735-6757(16)30677-5 [pii]

LID - 10.1016/j.ajem.2016.09.066 [doi]

AB - BACKGROUND/PURPOSE: To determine the impact of delayed admission to the intensive

care unit (ICU) on the clinical outcomes of patients with acute respiratory

failure (ARF) in the emergency department (ED). METHODS: This retrospective

cohort study included non-traumatic adult patients with ARF and mechanical

ventilation support in the ED of a tertiary university hospital in Taiwan from

January 1, 2013, to August 31, 2013. Clinical data were extracted from chart

records. The primary and secondary outcome measures were a prolonged hospital

stay (>30 days) and the in-hospital crude mortality within 90 days, respectively.

RESULTS: For 267 eligible patients (age range 21.0-98.0 years, mean 70.5±15.1

years; male 184, 68.9%), multivariate analysis was used to determine the

significant adverse effects of an ED stay >1.0 hour on in-hospital crude

mortality (odds ratio 2.19, P<.05), which was thus defined as delayed ICU

admission. In-hospital mortality significantly differed between patients with

delayed ICU admission and those without delayed admission, as revealed by the

Kaplan-Meier survival curves (P<.05). Moreover, a linear-by-linear correlation

was observed between the length of ICU waiting time in the ED and the lengths of

total hospital stay (r=0.152, P<.05), ICU stay (r=0.148, P<.05), and ventilator

support (r=0.222, P<.05). CONCLUSIONS: For patients with ARF who required

mechanical ventilation support and intensive care, a delayed ICU admission more

than 1.0 hour is a strong determinant of mortality and is associated with a

longer ICU stay and a longer need for ventilation.

CI - Copyright © 2016 Elsevier Inc. All rights reserved.

FAU - Hsieh, Chih-Chia

AU - Hsieh CC

AD - Department of Emergency Medicine, National Cheng Kung University Hospital,

College of Medicine, National Cheng Kung University, Tainan, Taiwan.

FAU - Lee, Ching-Chi

AU - Lee CC

AD - Department of Internal Medicine, Madou Sin-Lau Hospital, Tainan, Taiwan; Graduate

Institute of Medical Sciences, College of Health Sciences, Chang Jung Christian

University, Tainan, Taiwan.

FAU - Hsu, Hsiang-Chin

AU - Hsu HC

AD - Department of Emergency Medicine, National Cheng Kung University Hospital,

College of Medicine, National Cheng Kung University, Tainan, Taiwan.

FAU - Shih, Hsin-I

AU - Shih HI

AD - Department of Emergency Medicine, National Cheng Kung University Hospital,

College of Medicine, National Cheng Kung University, Tainan, Taiwan; Department

of Public Health, National Cheng Kung University Hospital, College of Medicine,

National Cheng Kung University, Tainan, Taiwan.

FAU - Lu, Chien-Hsin

AU - Lu CH

AD - Department of Emergency Medicine, National Cheng Kung University Hospital,

College of Medicine, National Cheng Kung University, Tainan, Taiwan.

FAU - Lin, Chih-Hao

AU - Lin CH

AD - Department of Emergency Medicine, National Cheng Kung University Hospital,

College of Medicine, National Cheng Kung University, Tainan, Taiwan. Electronic

address: emergency.lin@gmail.com.

LA - eng

PT - Journal Article

DEP - 20160930

PL - United States

TA - Am J Emerg Med

JT - The American journal of emergency medicine

JID - 8309942

SB - IM

CIN - Am J Emerg Med. 2017 Jun;35(6):914-915. PMID: 28318801

CIN - Am J Emerg Med. 2017 Oct;35(10):1571-1572. PMID: 28502761

CIN - Emerg Med Australas. 2023 Aug;35(4):612-617. PMID: 36718053

MH - Acute Disease

MH - Adult

MH - Aged

MH - Aged, 80 and over

MH - Cohort Studies

MH - Emergency Service, Hospital

MH - Female

MH - *Hospital Mortality

MH - *Hospitalization

MH - Humans

MH - *Intensive Care Units

MH - Length of Stay/*statistics & numerical data

MH - Male

MH - Middle Aged

MH - Multivariate Analysis

MH - Odds Ratio

MH - Respiration, Artificial

MH - Respiratory Insufficiency/mortality/*therapy

MH - Retrospective Studies

MH - Time Factors

MH - Young Adult

EDAT- 2016/10/16 06:00

MHDA- 2017/03/21 06:00

CRDT- 2016/10/16 06:00

PHST- 2016/05/27 00:00 [received]

PHST- 2016/08/31 00:00 [revised]

PHST- 2016/09/29 00:00 [accepted]

PHST- 2016/10/16 06:00 [pubmed]

PHST- 2017/03/21 06:00 [medline]

PHST- 2016/10/16 06:00 [entrez]

AID - S0735-6757(16)30677-5 [pii]

AID - 10.1016/j.ajem.2016.09.066 [doi]

PST - ppublish

SO - Am J Emerg Med. 2017 Jan;35(1):39-44. doi: 10.1016/j.ajem.2016.09.066. Epub 2016

Sep 30.

1. **PMID- 31575708**

OWN - NLM

STAT- MEDLINE

DCOM- 20201125

LR - 20201125

IS - 1943-3654 (Electronic)

IS - 0020-1324 (Print)

IS - 0020-1324 (Linking)

VI - 65

IP - 1

DP - 2020 Jan

TI - Noninvasive Ventilation for Critically Ill Subjects With Acute Respiratory

Failure in the Emergency Department.

PG - 82-90

LID - 10.4187/respcare.07111 [doi]

AB - BACKGROUND: We aimed to investigate the association between noninvasive

ventilation (NIV) initiated in the emergency department and patient outcomes for

those requiring invasive mechanical ventilation so that we could understand the

effect of extended NIV use (ie, > 4 h) prior to invasive mechanical ventilation

on patient outcomes. METHODS: We conducted a retrospective single-center cohort

study at an academic tertiary care hospital center. All emergency department

patients with acute respiratory failure requiring invasive mechanical ventilation

and admission to the ICU within 48 h of initial presentation over a 24-month

period were included. RESULTS: Subject characteristics, ventilator parameters,

and clinical course were captured via electronic query, respiratory billing data,

and standardized chart abstraction. A total of 431 subjects with acute

respiratory failure requiring invasive mechanical ventilation within 48 h of

arrival were identified, of whom 115 (26.7%) were exposed to NIV prior to

invasive mechanical ventilation, with a median duration of 4 h (interquartile

range 1.9-9.3). Based on a multivariable model controlling for covariates, any

NIV exposure prior to invasive mechanical ventilation was not associated with an

increased odds of persistent organ dysfunction or death. However, in the subset

of subjects exposed to NIV, extended NIV use (ie, > 4 h) prior to invasive

mechanical ventilation was associated with increased odds of persistent organ

dysfunction or death (odds ratio 4.11, 95% CI 1.51-11.19). Extended NIV use was

also associated with increased odds of in-hospital mortality (odds ratio 4.02,

95% CI 1.51-10.74). CONCLUSIONS: Although any exposure to NIV prior to invasive

mechanical ventilation did not appear to affect morbidity and mortality, extended

NIV use prior to invasive mechanical ventilation was associated with worse

patient outcomes, suggesting a need for additional study to better understand the

ramifications of duration of NIV use prior to failure on outcomes. Given this

early timeframe for intervention, future studies should be collaborations between

the emergency department and ICU.

CI - Copyright © 2020 by Daedalus Enterprises.

FAU - Goel, Neha N

AU - Goel NN

AD - Division of Pulmonary, Critical Care, and Sleep Medicine, Department of Medicine,

Icahn School of Medicine at Mount Sinai, New York, New York.

neha.goel@mountsinai.org.

FAU - Owyang, Clark

AU - Owyang C

AD - Department of Emergency Medicine, Icahn School of Medicine at Mount Sinai, New

York, New York.

FAU - Ranginwala, Shamsuddoha

AU - Ranginwala S

AD - Department of Respiratory Therapy, Icahn School of Medicine at Mount Sinai, New

York, New York.

FAU - Loo, George T

AU - Loo GT

AD - Department of Emergency Medicine, Icahn School of Medicine at Mount Sinai, New

York, New York.

AD - Department of Population Health Science and Policy, Icahn School of Medicine at

Mount Sinai, New York, New York.

FAU - Richardson, Lynne D

AU - Richardson LD

AD - Department of Emergency Medicine, Icahn School of Medicine at Mount Sinai, New

York, New York.

AD - Department of Population Health Science and Policy, Icahn School of Medicine at

Mount Sinai, New York, New York.

FAU - Mathews, Kusum S

AU - Mathews KS

AD - Division of Pulmonary, Critical Care, and Sleep Medicine, Department of Medicine,

Icahn School of Medicine at Mount Sinai, New York, New York.

LA - eng

GR - K23 HL130648/HL/NHLBI NIH HHS/United States

PT - Journal Article

DEP - 20191001

PL - United States

TA - Respir Care

JT - Respiratory care

JID - 7510357

SB - IM

MH - Cohort Studies

MH - Critical Illness

MH - Emergency Service, Hospital

MH - Hospital Mortality

MH - Humans

MH - Intensive Care Units

MH - Intubation/*mortality

MH - Noninvasive Ventilation/*mortality

MH - Respiration, Artificial/*mortality

MH - Respiratory Insufficiency/*therapy

MH - Retrospective Studies

MH - Treatment Failure

PMC - PMC7119184

OTO - NOTNLM

OT - bi-level

OT - critically ill

OT - emergency department

OT - mechanical ventilation

OT - noninvasive ventilation

OT - respiratory failure

COIS- Dr Goel presented a version of this paper was presented at the American Thoracic

Society 2018 International Conference, held May 18-23, 2018, in San Diego,

California. Dr Goel is partially supported by National Institutes of Health (NIH)

National Heart, Lung, and Blood Institute Award DHHS – 1T32 HL129974-PI:

Richardson. Dr Mathews is partially supported by NIH National Heart, Lung, and

Blood Institute Award 1K23HL130648-PI: Mathews. This work was supported in part

through the Mount Sinai Data Warehouse resources and staff expertise provided by

Scientific Computing at the Icahn School of Medicine at Mount Sinai. The other

authors have disclosed no conflicts of interest.

EDAT- 2019/10/03 06:00

MHDA- 2020/11/26 06:00

CRDT- 2019/10/03 06:00

PHST- 2019/10/03 06:00 [pubmed]

PHST- 2020/11/26 06:00 [medline]

PHST- 2019/10/03 06:00 [entrez]

AID - respcare.07111 [pii]

AID - RC-07111 [pii]

AID - 10.4187/respcare.07111 [doi]

PST - ppublish

SO - Respir Care. 2020 Jan;65(1):82-90. doi: 10.4187/respcare.07111. Epub 2019 Oct 1.

1. **PMID- 35895629**

OWN - NLM

STAT- MEDLINE

DCOM- 20230303

LR - 20230310

IS - 2325-6621 (Electronic)

IS - 2329-6933 (Print)

IS - 2325-6621 (Linking)

VI - 20

IP - 3

DP - 2023 Mar

TI - Among-Hospital Variation in Intensive Care Unit Admission Practices and

Associated Outcomes for Patients with Acute Respiratory Failure.

PG - 406-413

LID - 10.1513/AnnalsATS.202205-429OC [doi]

AB - Rationale: We have previously shown that hospital strain is associated with

intensive care unit (ICU) admission and that ICU admission, compared with ward

admission, may benefit certain patients with acute respiratory failure (ARF).

Objectives: To understand how strain-process-outcomes relationships in patients

with ARF may vary among hospitals and what hospital practice differences may

account for such variation. Methods: We examined high-acuity patients with ARF

who did not require mechanical ventilation or vasopressors in the emergency

department (ED) and were admitted to 27 U.S. hospitals from 2013 to 2018.

Stratifying by hospital, we compared hospital strain-ICU admission relationships

and hospital length of stay (LOS) and mortality among patients initially admitted

to the ICU versus the ward using hospital strain as a previously validated

instrumental variable. We also surveyed hospital practices and, in exploratory

analyses, evaluated their associations with the above processes and outcomes.

Results: There was significant among-hospital variation in ICU admission rates,

in hospital strain-ICU admission relationships, and in the association of ICU

admission with hospital LOS and hospital mortality. Overall, ED patients with ARF

(n = 45,339) experienced a 0.82-day shorter median hospital LOS if admitted

initially to the ICU compared with the ward, but among the 27 hospitals

(n = 224-3,324), this effect varied from 5.85 days shorter (95% confidence

interval [CI], -8.84 to -2.86; P < 0.001) to 4.38 days longer (95% CI, 1.86-6.90;

P = 0.001). Corresponding ranges for in-hospital mortality with ICU compared with

ward admission revealed odds ratios from 0.08 (95% CI, 0.01-0.56; P < 0.007) to

8.89 (95% CI, 1.60-79.85; P = 0.016) among patients with ARF (pooled odds ratio,

0.75). In exploratory analyses, only a small number of measured hospital

practices-the presence of a sepsis ED disposition guideline and maximum ED

patient capacity-were potentially associated with hospital strain-ICU admission

relationships. Conclusions: Hospitals vary considerably in ICU admission rates,

the sensitivity of those rates to hospital capacity strain, and the benefits of

ICU admission for patients with ARF not requiring life support therapies in the

ED. Future work is needed to more fully identify hospital-level factors

contributing to these relationships.

FAU - Anesi, George L

AU - Anesi GL

AUID- ORCID: 0000-0003-4585-0714

AD - Division of Pulmonary, Allergy, and Critical Care, Perelman School of Medicine.

AD - Leonard Davis Institute of Health Economics.

AD - Palliative and Advanced Illness Research Center, Perelman School of Medicine.

FAU - Dress, Erich

AU - Dress E

AD - Palliative and Advanced Illness Research Center, Perelman School of Medicine.

FAU - Chowdhury, Marzana

AU - Chowdhury M

AD - Palliative and Advanced Illness Research Center, Perelman School of Medicine.

FAU - Wang, Wei

AU - Wang W

AD - Palliative and Advanced Illness Research Center, Perelman School of Medicine.

FAU - Small, Dylan S

AU - Small DS

AD - Department of Statistics, The Wharton School.

FAU - Delgado, M Kit

AU - Delgado MK

AD - Leonard Davis Institute of Health Economics.

AD - Palliative and Advanced Illness Research Center, Perelman School of Medicine.

AD - Center for Emergency Care Policy and Research, Department of Emergency Medicine,

Perelman School of Medicine, and.

FAU - Bayes, Brian

AU - Bayes B

AD - Palliative and Advanced Illness Research Center, Perelman School of Medicine.

FAU - Szymczak, Julia E

AU - Szymczak JE

AD - Department of Biostatistics, Epidemiology, and Informatics, Perelman School of

Medicine, University of Pennsylvania, Philadelphia, Pennsylvania; and.

FAU - Glassman, Lindsay W

AU - Glassman LW

AD - Department of Biostatistics, Epidemiology, and Informatics, Perelman School of

Medicine, University of Pennsylvania, Philadelphia, Pennsylvania; and.

FAU - Barreda, Fernando X

AU - Barreda FX

AD - Division of Research, Kaiser Permanente, Oakland, California.

FAU - Weiner, Jonathan Z

AU - Weiner JZ

AD - Division of Research, Kaiser Permanente, Oakland, California.

FAU - Escobar, Gabriel J

AU - Escobar GJ

AD - Division of Research, Kaiser Permanente, Oakland, California.

FAU - Halpern, Scott D

AU - Halpern SD

AUID- ORCID: 0000-0002-3603-4769

AD - Division of Pulmonary, Allergy, and Critical Care, Perelman School of Medicine.

AD - Leonard Davis Institute of Health Economics.

AD - Palliative and Advanced Illness Research Center, Perelman School of Medicine.

AD - Department of Biostatistics, Epidemiology, and Informatics, Perelman School of

Medicine, University of Pennsylvania, Philadelphia, Pennsylvania; and.

FAU - Liu, Vincent X

AU - Liu VX

AD - Division of Research, Kaiser Permanente, Oakland, California.

LA - eng

GR - K23 HL161353/HL/NHLBI NIH HHS/United States

GR - L30 HL138827/HL/NHLBI NIH HHS/United States

GR - L30 HL154200/HL/NHLBI NIH HHS/United States

GR - R01 HL136719/HL/NHLBI NIH HHS/United States

PT - Journal Article

PT - Research Support, N.I.H., Extramural

PL - United States

TA - Ann Am Thorac Soc

JT - Annals of the American Thoracic Society

JID - 101600811

SB - IM

CIN - Ann Am Thorac Soc. 2023 Mar;20(3):364-366. PMID: 36856718

MH - Humans

MH - Hospitalization

MH - Length of Stay

MH - Intensive Care Units

MH - Emergency Service, Hospital

MH - Hospitals

MH - Hospital Mortality

MH - *Respiratory Distress Syndrome

MH - *Respiratory Insufficiency/therapy

MH - Retrospective Studies

PMC - PMC9993147

OTO - NOTNLM

OT - acute respiratory failure

OT - hospital strain

OT - hospital variation

OT - intensive care unit

OT - processes of care

EDAT- 2022/07/28 06:00

MHDA- 2023/03/04 06:00

PMCR- 2024/03/01

CRDT- 2022/07/27 13:34

PHST- 2024/03/01 00:00 [pmc-release]

PHST- 2022/07/28 06:00 [pubmed]

PHST- 2023/03/04 06:00 [medline]

PHST- 2022/07/27 13:34 [entrez]

AID - 10.1513/AnnalsATS.202205-429OC [doi]

PST - ppublish

SO - Ann Am Thorac Soc. 2023 Mar;20(3):406-413. doi: 10.1513/AnnalsATS.202205-429OC.

1. **PMID- 26210474**

OWN - NLM

STAT- MEDLINE

DCOM- 20160711

LR - 20181202

IS - 1432-1750 (Electronic)

IS - 0341-2040 (Linking)

VI - 193

IP - 5

DP - 2015 Oct

TI - Where is Noninvasive Ventilation Actually Delivered for Acute Respiratory

Failure?

PG - 779-88

LID - 10.1007/s00408-015-9766-y [doi]

AB - PURPOSE: Few studies have examined locations of noninvasive ventilation (NIV)

application for acute respiratory failure (ARF). We aimed to track actual

locations of NIV delivery and related outcomes. METHODS: Observational cohort

study based at 8 acute care hospitals in Massachusetts on adult patients admitted

for ARF requiring ventilatory support during pre-determined time intervals.

RESULTS: Of 1225 ventilator starts, 499 were NIV; 209 (42%) in intensive care

units (ICU), 185 (37%) in emergency departments (ED), 91 (18%) on general wards,

and 14 (3%) in other units. Utilization (% of all ventilator starts) (1), success

(2) and in-hospital mortality (3) rates for patients initiated on NIV in ICU, ED,

and general and other wards were (1) 38, 36, 73, and 52%, (2) 60, 77, 68, and 93%

and (3) 25, 12, 17, and 0%, respectively (p < 0.05 for all). Patients with

acute-on-chronic lung disease (ACLD) and acute pulmonary edema (APE) were begun

on NIV most often in EDs and patients with 'de novo' ARF and neurologic disorders

most often in ICU's. Approximately 2/3 of patients begun on NIV outside of ICUs

were transferred within 72 h to ICUs, wards or other units. CONCLUSIONS: Most NIV

starts occurred in ICUs and EDs but utilization rate was highest (>50%) on

general wards where a fifth of NIV starts took place. Actual location depended on

etiology of ARF as patients with ACLD and APE were started more often in EDs and

"de novo" ARF in ICU. NIV failure and mortality rates were higher in ICUs related

to the greater proportion of patients with "de novo" ARF.

FAU - Ozsancak Ugurlu, Aylin

AU - Ozsancak Ugurlu A

AD - Department of Pulmonary Disease, Baskent University, Oymaci sok. No: 2, 34662,

Altunizade/Istanbul, Turkey. aozsancak@hotmail.com.

FAU - Sidhom, Samy S

AU - Sidhom SS

AD - Pulmonary Department, Newton-Wellesley Hospital, Newton, MA, USA.

FAU - Khodabandeh, Ali

AU - Khodabandeh A

AD - Mass Lung and Allergy PC, Leominster, MA, USA.

FAU - Ieong, Michael

AU - Ieong M

AD - Boston Medical Center, Boston, MA, USA.

FAU - Mohr, Chester

AU - Mohr C

AD - Cape Cod Health Systems, Hyannis, MA, USA.

FAU - Lin, Denis Y

AU - Lin DY

AD - Lowell General Hospital, Lowell, MA, USA.

FAU - Buchwald, Irwin

AU - Buchwald I

AD - Saints Medical Center, Lowell, MA, USA.

FAU - Bahhady, Imad

AU - Bahhady I

AD - Morton Hospital, Taunton, MA, USA.

FAU - Wengryn, John

AU - Wengryn J

AD - Jordan Hospital, Plymouth, MA, USA.

FAU - Maheshwari, Vinay

AU - Maheshwari V

AD - Pulmonary Associates, Newark, DE, USA.

FAU - Hill, Nicholas S

AU - Hill NS

AD - Department of Pulmonary, Critical Care and Sleep Medicine, Tufts Medical Center,

Boston, MA, USA.

LA - eng

SI - ClinicalTrials.gov/NCT00458926

PT - Journal Article

PT - Observational Study

PT - Research Support, Non-U.S. Gov't

DEP - 20150726

PL - United States

TA - Lung

JT - Lung

JID - 7701875

SB - IM

MH - Acute Disease

MH - Aged

MH - Aged, 80 and over

MH - Chronic Disease

MH - Emergency Service, Hospital/*statistics & numerical data

MH - Female

MH - Hospital Mortality

MH - Hospitals

MH - Humans

MH - Intensive Care Units/*statistics & numerical data

MH - Male

MH - Massachusetts

MH - Middle Aged

MH - Nervous System Diseases/complications

MH - Noninvasive Ventilation/*statistics & numerical data

MH - Patients' Rooms/*statistics & numerical data

MH - Pulmonary Edema/complications/therapy

MH - Respiratory Insufficiency/etiology/*therapy

MH - Treatment Outcome

OTO - NOTNLM

OT - Acute respiratory failure

OT - Emergency care

OT - General wards

OT - Intensive care units

OT - Noninvasive ventilation

EDAT- 2015/07/27 06:00

MHDA- 2016/07/12 06:00

CRDT- 2015/07/27 06:00

PHST- 2015/01/29 00:00 [received]

PHST- 2015/07/19 00:00 [accepted]

PHST- 2015/07/27 06:00 [entrez]

PHST- 2015/07/27 06:00 [pubmed]

PHST- 2016/07/12 06:00 [medline]

AID - 10.1007/s00408-015-9766-y [pii]

AID - 10.1007/s00408-015-9766-y [doi]

PST - ppublish

SO - Lung. 2015 Oct;193(5):779-88. doi: 10.1007/s00408-015-9766-y. Epub 2015 Jul 26.

1. **PMID- 23185988**

OWN - NLM

STAT- MEDLINE

DCOM- 20140224

LR - 20190116

IS - 1029-2403 (Electronic)

IS - 1026-8022 (Linking)

VI - 54

IP - 8

DP - 2013 Aug

TI - Delayed intensive care unit admission is associated with increased mortality in

patients with cancer with acute respiratory failure.

PG - 1724-9

LID - 10.3109/10428194.2012.753446 [doi]

AB - Acute respiratory failure (ARF) is the leading reason for intensive care unit

(ICU) admission in patients with cancer. The aim of this study was to identify

early predictors of death in patients with cancer admitted to the ICU for ARF who

were not intubated at admission. We conducted analysis of a prospective

randomized controlled trial including 219 patients with cancer with ARF in which

day-28 mortality was a secondary endpoint. Mortality at day 28 was 31.1%. By

multivariate analysis, independent predictors of day-28 mortality were: age (odds

ratio [OR] 1.30/10 years, 95% confidence interval [CI] [1.01-1.68], p = 0.04),

more than one line of chemotherapy (OR 2.14, 95% CI [1.08-4.21], p = 0.03), time

between respiratory symptoms onset and ICU admission > 2 days (OR 2.50, 95% CI

[1.25-5.02], p = 0.01), oxygen flow at admission (OR 1.07/L, 95% CI [1.00-1.14],

p = 0.04) and extra-respiratory symptoms (OR 2.84, 95%CI [1.30-6.21], p = 0.01).

After adjustment for the logistic organ dysfunction (LOD) score at admission,

only time between respiratory symptoms onset and ICU admission > 2 days and LOD

score were independently associated with day-28 mortality. Determinants of death

include both factors non-amenable to change, and delay in ARF management. These

results suggest that early intensive care management of patients with cancer with

ARF may translate to better survival.

FAU - Mokart, Djamel

AU - Mokart D

AD - Réanimation Polyvalente, Institut Paoli-Calmettes, Marseille, France.

mokartd@ipc.unicancer.fr

FAU - Lambert, Jérôme

AU - Lambert J

FAU - Schnell, David

AU - Schnell D

FAU - Fouché, Louis

AU - Fouché L

FAU - Rabbat, Antoine

AU - Rabbat A

FAU - Kouatchet, Achille

AU - Kouatchet A

FAU - Lemiale, Virginie

AU - Lemiale V

FAU - Vincent, François

AU - Vincent F

FAU - Lengliné, Etienne

AU - Lengliné E

FAU - Bruneel, Fabrice

AU - Bruneel F

FAU - Pene, Frederic

AU - Pene F

FAU - Chevret, Sylvie

AU - Chevret S

FAU - Azoulay, Elie

AU - Azoulay E

LA - eng

PT - Journal Article

DEP - 20121226

PL - United States

TA - Leuk Lymphoma

JT - Leukemia & lymphoma

JID - 9007422

SB - IM

MH - Aged

MH - Female

MH - Hospital Mortality

MH - Humans

MH - *Intensive Care Units

MH - Male

MH - Middle Aged

MH - Neoplasms/*complications/*mortality

MH - *Patient Admission

MH - Prognosis

MH - Prospective Studies

MH - Respiratory Insufficiency/*complications/*mortality

EDAT- 2012/11/29 06:00

MHDA- 2014/02/25 06:00

CRDT- 2012/11/29 06:00

PHST- 2012/11/29 06:00 [entrez]

PHST- 2012/11/29 06:00 [pubmed]

PHST- 2014/02/25 06:00 [medline]

AID - 10.3109/10428194.2012.753446 [doi]

PST - ppublish

SO - Leuk Lymphoma. 2013 Aug;54(8):1724-9. doi: 10.3109/10428194.2012.753446. Epub

2012 Dec 26.

1. **PMID- 32389397**

OWN - NLM

STAT- MEDLINE

DCOM- 20200824

LR - 20201210

IS - 1532-8171 (Electronic)

IS - 0735-6757 (Linking)

VI - 38

IP - 7

DP - 2020 Jul

TI - High-flow nasal cannula oxygen therapy in acute respiratory failure at Emergency

Departments: A systematic review.

PG - 1508-1514

LID - S0735-6757(20)30323-5 [pii]

LID - 10.1016/j.ajem.2020.04.091 [doi]

AB - OBJECTIVES: The use of high-flow oxygen therapy (HFOT) through nasal cannula for

the management of acute respiratory failure at the emergency department (ED) has

been only sparsely studied. We conducted a systematic review of

randomized-controlled and quasi-experimental studies comparing the early use of

HFOT versus conventional oxygen therapy (COT) in patients with acute respiratory

failure admitted to EDs. METHODS: A systematic research of literature was carried

out for all published control trials comparing HFOT with COT in adult patients

admitted in EDs. Eligible data were extracted from Medline, Embase, Pascal, Web

of Science and the Cochrane database. The primary outcome was the need for

mechanical ventilation, i.e. intubation or non-invasive ventilation as rescue

therapy. Secondary outcomes included respiratory rate, dyspnea level, ED length

of stay, intubation and mortality. RESULTS: Out of 1829 studies screened, five

studies including 673 patients were retained in the analysis (350 patients

treated with HFOT and 323 treated with COT). The need for mechanical ventilation

was similar in both treatments (RR = 0.75; 95% CI 0.41 to 1.35; P = 0.31;

I(2) = 16%). Respiratory rate was lower with HFOT (Mean difference (MD) = -3.14

breaths/min; 95% CI = -4.9 to -1.4; P < 0.001; I(2) = 39%), whereas sensation of

dyspnea did not differ. (MD = -1.04; 95% CI = -2.29 to -0.22; P = 0.08;

I(2) = 67%). ED length of stay and mortality were similar between groups.

CONCLUSION: The early use of HFOT in patients admitted to an ED for acute

respiratory failure did not reduce the need for mechanical ventilation as

compared to COT. However, HFOT decreased respiratory rate. REGISTRATION: PROSPERO

ID CRD42019125696.

CI - Copyright © 2020. Published by Elsevier Inc.

FAU - Marjanovic, Nicolas

AU - Marjanovic N

AD - CHU de Poitiers, Service des Urgences et SAMU 86, Poitiers, France; INSERM,

CIC-1402 Team 5 ALIVE, Poitiers, France; Université de Poitiers, Faculté de

Médecine et de Pharmacie de Poitiers, Poitiers, France. Electronic address:

marjanovic@chu-poitiers.fr.

FAU - Guénézan, Jérémy

AU - Guénézan J

AD - CHU de Poitiers, Service des Urgences et SAMU 86, Poitiers, France; Université de

Poitiers, Faculté de Médecine et de Pharmacie de Poitiers, Poitiers, France.

FAU - Frat, Jean-Pierre

AU - Frat JP

AD - INSERM, CIC-1402 Team 5 ALIVE, Poitiers, France; Université de Poitiers, Faculté

de Médecine et de Pharmacie de Poitiers, Poitiers, France; CHU de Poitiers,

Médecine Intensive Réanimation, Poitiers, France.

FAU - Mimoz, Olivier

AU - Mimoz O

AD - CHU de Poitiers, Service des Urgences et SAMU 86, Poitiers, France; Université de

Poitiers, Faculté de Médecine et de Pharmacie de Poitiers, Poitiers, France.

FAU - Thille, Arnaud W

AU - Thille AW

AD - INSERM, CIC-1402 Team 5 ALIVE, Poitiers, France; Université de Poitiers, Faculté

de Médecine et de Pharmacie de Poitiers, Poitiers, France; CHU de Poitiers,

Médecine Intensive Réanimation, Poitiers, France.

LA - eng

PT - Journal Article

PT - Systematic Review

DEP - 20200504

PL - United States

TA - Am J Emerg Med

JT - The American journal of emergency medicine

JID - 8309942

SB - IM

MH - Dyspnea/*therapy

MH - Emergency Service, Hospital

MH - Hospital Mortality

MH - Humans

MH - Length of Stay

MH - Oxygen Inhalation Therapy/*methods

MH - Respiration, Artificial/statistics & numerical data

MH - Respiratory Distress Syndrome/*therapy

MH - Respiratory Insufficiency/*therapy

MH - Respiratory Rate

OTO - NOTNLM

OT - Emergency department

OT - High-flow nasal cannula

OT - Respiratory failure

COIS- Declaration of competing interest NM reports travel expense coverage to attend

scientific meetings and payment for lectures from Fisher & Paykel. JPF reports

travel expense coverage to attend scientific meetings and personal fees from

Fisher & Paykel. AWT reports travel expense coverage to attend scientific

meetings and payment for lectures from Fisher & Paykel.

EDAT- 2020/05/12 06:00

MHDA- 2020/08/25 06:00

CRDT- 2020/05/12 06:00

PHST- 2020/01/26 00:00 [received]

PHST- 2020/04/17 00:00 [revised]

PHST- 2020/04/26 00:00 [accepted]

PHST- 2020/05/12 06:00 [pubmed]

PHST- 2020/08/25 06:00 [medline]

PHST- 2020/05/12 06:00 [entrez]

AID - S0735-6757(20)30323-5 [pii]

AID - 10.1016/j.ajem.2020.04.091 [doi]

PST - ppublish

SO - Am J Emerg Med. 2020 Jul;38(7):1508-1514. doi: 10.1016/j.ajem.2020.04.091. Epub

2020 May 4.

1. **PMID- 23650433**

OWN - NLM

STAT- MEDLINE

DCOM- 20140818

LR - 20191210

IS - 1943-3654 (Electronic)

IS - 0020-1324 (Linking)

VI - 58

IP - 12

DP - 2013 Dec

TI - Influence of the admission pattern on the outcome of patients admitted to a

respiratory intensive care unit: does a step-down admission differ from a step-up

one?

PG - 2053-60

LID - 10.4187/respcare.02225 [doi]

AB - BACKGROUND: The outcomes of patients admitted to a respiratory ICU (RICU) have

been evaluated in the past, but no study has considered the influence of location

prior to RICU admission. METHODS: We analyzed prospectively collected data from

326 consecutive patients admitted to a 7-bed RICU. The primary end points were

survival and severity of morbidity-related complications, evaluated according to

the patient's location prior to RICU admission. Three admission pathways were

considered: step-down for patients transferred from the ICUs of our hospital;

step-up for patients coming from our respiratory wards or other medical wards;

and directly for patients coming from the emergency department. The secondary end

point was the potential influence of several risk factors for morbidity and

mortality. RESULTS: Of the 326 subjects, 92 (28%) died. Overall, subjects

admitted in a step-up process had a significantly higher mortality (P < .001)

than subjects in the other groups. The mortality rate was 64% for subjects

admitted from respiratory ward, 43% for those from medical wards, and 18% for

subjects from both ICU and emergency department (respiratory ward vs medical ward

P = .04, respiratory ward vs emergency department P < .001, respiratory ward vs

ICU P < .001, medical ward vs emergency department P < .001, and medical ward vs

ICU P < .001). Subjects admitted from a respiratory ward had a lower albumin

level, and Simplified Acute Physiology Score II was significantly higher in

subjects following a step-up admission. About 30% of the subjects admitted from a

respiratory ward received noninvasive ventilation as a "ceiling treatment." The

highest odds ratios related to survival were subject location prior to RICU

admission and female sex. Lack of use of noninvasive ventilation, younger age,

female sex, higher albumin level, lower Simplified Acute Physiology Score II,

higher Barthel score, and absence of chronic heart failure were also

statistically associated with a lower risk of death. CONCLUSIONS: The pathway of

admission to a RICU is a determinant of outcome. Patients following a step-up

pattern are more likely to die. Other major determinants of survival are age,

nutritional status and female sex.

FAU - Valentini, Ilaria

AU - Valentini I

AD - Department of Specialist, Diagnostic and Experimental Medicine.

FAU - Pacilli, Angela Maria Grazia

AU - Pacilli AM

FAU - Carbonara, Paolo

AU - Carbonara P

FAU - Fasano, Luca

AU - Fasano L

FAU - Vitale, Rita

AU - Vitale R

FAU - Zenesini, Corrado

AU - Zenesini C

FAU - Melotti, Rita Maria

AU - Melotti RM

FAU - Faenza, Stefano

AU - Faenza S

FAU - Nava, Stefano

AU - Nava S

LA - eng

PT - Journal Article

DEP - 20130430

PL - United States

TA - Respir Care

JT - Respiratory care

JID - 7510357

SB - IM

MH - APACHE

MH - Adult

MH - Aged

MH - Aged, 80 and over

MH - Critical Pathways/standards

MH - *Emergency Medical Services/methods/statistics & numerical data

MH - Emergency Service, Hospital/*statistics & numerical data

MH - Female

MH - Humans

MH - Italy/epidemiology

MH - Male

MH - Middle Aged

MH - Organizational Policy

MH - Outcome and Process Assessment, Health Care

MH - *Patient Admission/standards/statistics & numerical data/trends

MH - Prospective Studies

MH - Respiratory Care Units/statistics & numerical data

MH - *Respiratory Insufficiency/etiology/mortality/therapy

MH - Risk Assessment

MH - Risk Factors

MH - Survival Analysis

MH - Transportation of Patients/*methods

OTO - NOTNLM

OT - ICU

OT - acute respiratory failure

OT - critically ill patient

OT - noninvasive ventilation

OT - respiratory ICU

OT - weaning

EDAT- 2013/05/08 06:00

MHDA- 2014/08/19 06:00

CRDT- 2013/05/08 06:00

PHST- 2013/05/08 06:00 [entrez]

PHST- 2013/05/08 06:00 [pubmed]

PHST- 2014/08/19 06:00 [medline]

AID - respcare.02225 [pii]

AID - 10.4187/respcare.02225 [doi]

PST - ppublish

SO - Respir Care. 2013 Dec;58(12):2053-60. doi: 10.4187/respcare.02225. Epub 2013 Apr

30.

1. **PMID- 31393321**

OWN - NLM

STAT- MEDLINE

DCOM- 20200525

LR - 20200525

IS - 1530-0293 (Electronic)

IS - 0090-3493 (Print)

IS - 0090-3493 (Linking)

VI - 47

IP - 11

DP - 2019 Nov

TI - Emergency Department to ICU Time Is Associated With Hospital Mortality: A

Registry Analysis of 14,788 Patients From Six University Hospitals in The

Netherlands.

PG - 1564-1571

LID - 10.1097/CCM.0000000000003957 [doi]

AB - OBJECTIVES: Prolonged emergency department to ICU waiting time may delay

intensive care treatment, which could negatively affect patient outcomes. The aim

of this study was to investigate whether emergency department to ICU time is

associated with hospital mortality. DESIGN, SETTING, AND PATIENTS: We conducted a

retrospective observational cohort study using data from the Dutch quality

registry National Intensive Care Evaluation. Adult patients admitted to the ICU

directly from the emergency department in six university hospitals, between 2009

and 2016, were included. Using a logistic regression model, we investigated the

crude and adjusted (for disease severity; Acute Physiology and Chronic Health

Evaluation IV probability) odds ratios of emergency department to ICU time on

mortality. In addition, we assessed whether the Acute Physiology and Chronic

Health Evaluation IV probability modified the effect of emergency department to

ICU time on mortality. Secondary outcomes were ICU, 30-day, and 90-day mortality.

INTERVENTIONS: None. MEASUREMENTS AND MAIN RESULTS: A total of 14,788 patients

were included. The median emergency department to ICU time was 2.0 hours

(interquartile range, 1.3-3.3 hr). Emergency department to ICU time was

correlated to adjusted hospital mortality (p < 0.002), in particular in patients

with the highest Acute Physiology and Chronic Health Evaluation IV probability

and long emergency department to ICU time quintiles: odds ratio, 1.29; 95% CI,

1.02-1.64 (2.4-3.7 hr) and odds ratio, 1.54; 95% CI, 1.11-2.14 (> 3.7 hr), both

compared with the reference category (< 1.2 hr). For 30-day and 90-day mortality,

we found similar results. However, emergency department to ICU time was not

correlated to adjusted ICU mortality (p = 0.20). CONCLUSIONS: Prolonged emergency

department to ICU time (> 2.4 hr) is associated with increased hospital mortality

after ICU admission, mainly driven by patients who had a higher Acute Physiology

and Chronic Health Evaluation IV probability. We hereby provide evidence that

rapid admission of the most critically ill patients to the ICU might reduce

hospital mortality.

FAU - Groenland, Carline N L

AU - Groenland CNL

AD - Department of Intensive Care Medicine, Erasmus MC, University Medical Center,

Rotterdam, The Netherlands.

FAU - Termorshuizen, Fabian

AU - Termorshuizen F

AD - Department of Medical Informatics, Amsterdam University Medical Center,

Amsterdam, The Netherlands.

AD - National Intensive Care Evaluation (NICE) foundation, Amsterdam, The Netherlands.

FAU - Rietdijk, Wim J R

AU - Rietdijk WJR

AD - Department of Intensive Care Medicine, Erasmus MC, University Medical Center,

Rotterdam, The Netherlands.

FAU - van den Brule, Judith

AU - van den Brule J

AD - Department of Intensive Care Medicine, Radboud University Medical Center,

Nijmegen, The Netherlands.

FAU - Dongelmans, Dave A

AU - Dongelmans DA

AD - Department of Intensive Care Medicine, Amsterdam University Medical Center,

Amsterdam, The Netherlands.

FAU - de Jonge, Evert

AU - de Jonge E

AD - Department of Intensive Care Medicine, University Medical Center Leiden, Leiden,

The Netherlands.

FAU - de Lange, Dylan W

AU - de Lange DW

AD - Department of Intensive Care Medicine, University Medical Center Utrecht,

Utrecht, The Netherlands.

FAU - de Smet, Anne Marie G A

AU - de Smet AMGA

AD - Department of Intensive Care Medicine, University Medical Center Groningen,

Groningen, The Netherlands.

FAU - de Keizer, Nicolette F

AU - de Keizer NF

AD - Department of Medical Informatics, Amsterdam University Medical Center,

Amsterdam, The Netherlands.

AD - National Intensive Care Evaluation (NICE) foundation, Amsterdam, The Netherlands.

FAU - Weigel, Joachim D

AU - Weigel JD

AD - Department of Intensive Care Medicine, Erasmus MC, University Medical Center,

Rotterdam, The Netherlands.

FAU - Jewbali, Lucia S D

AU - Jewbali LSD

AD - Department of Intensive Care Medicine, Erasmus MC, University Medical Center,

Rotterdam, The Netherlands.

AD - Department of Cardiology, Erasmus MC, University Medical Center, Rotterdam, The

Netherlands.

FAU - Boersma, Eric

AU - Boersma E

AD - Department of Cardiology, Erasmus MC, University Medical Center, Rotterdam, The

Netherlands.

FAU - den Uil, Corstiaan A

AU - den Uil CA

AD - Department of Intensive Care Medicine, Erasmus MC, University Medical Center,

Rotterdam, The Netherlands.

AD - Department of Cardiology, Erasmus MC, University Medical Center, Rotterdam, The

Netherlands.

LA - eng

PT - Journal Article

PT - Observational Study

PL - United States

TA - Crit Care Med

JT - Critical care medicine

JID - 0355501

SB - IM

CIN - Crit Care Med. 2019 Nov;47(11):1664-1665. PMID: 31609265

CIN - Crit Care Med. 2020 Mar;48(3):e252-e253. PMID: 32058394

CIN - Crit Care Med. 2020 Mar;48(3):e253-e254. PMID: 32058395

CIN - Crit Care Med. 2020 Mar;48(3):e254. PMID: 32058396

MH - APACHE

MH - Adult

MH - Aged

MH - Cohort Studies

MH - *Emergency Service, Hospital

MH - Female

MH - Heart Arrest/mortality

MH - Hematoma, Subdural/mortality

MH - *Hospital Mortality

MH - Hospitals, University

MH - Humans

MH - *Intensive Care Units

MH - Intracranial Hemorrhages/mortality

MH - Male

MH - Middle Aged

MH - Netherlands/epidemiology

MH - *Patient Admission

MH - Registries

MH - Respiratory Insufficiency/mortality

MH - Retrospective Studies

MH - Time Factors

MH - Wounds and Injuries/mortality

PMC - PMC6798749

EDAT- 2019/08/09 06:00

MHDA- 2020/05/26 06:00

CRDT- 2019/08/09 06:00

PHST- 2019/08/09 06:00 [pubmed]

PHST- 2020/05/26 06:00 [medline]

PHST- 2019/08/09 06:00 [entrez]

AID - 10.1097/CCM.0000000000003957 [doi]

PST - ppublish

SO - Crit Care Med. 2019 Nov;47(11):1564-1571. doi: 10.1097/CCM.0000000000003957.

1. **PMID- 24504638**

OWN - NLM

STAT- MEDLINE

DCOM- 20180117

LR - 20211021

IS - 1432-1238 (Electronic)

IS - 0342-4642 (Linking)

VI - 40

IP - 3

DP - 2014 Mar

TI - Is the volume of mechanically ventilated admissions to UK critical care units

associated with improved outcomes?

PG - 353-60

LID - 10.1007/s00134-013-3205-4 [doi]

AB - BACKGROUND: It is unknown whether a volume-outcome relationship exists for

mechanically ventilated admissions to UK critical care units. This study was

conducted to evaluate the volume-outcome relationship for mechanically ventilated

admissions to adult, general critical care units in the UK with a view to

informing policy, service delivery and organisation of specialist, advanced

respiratory care. METHODS: A retrospective cohort study using data from the Case

Mix Programme Database was conducted. The primary exposure of interest was annual

volume (absolute number) of mechanically ventilated admissions per critical care

unit per year. The primary outcome was ultimate acute hospital mortality. A

multivariable analysis was performed to assess the relationship between annual

volume and outcome while adjusting for a priori selected confounders. Two

interaction tests were performed. The first interaction test was between annual

volume and admission type and the second between annual volume and initial acute

severity of respiratory failure. Sensitivity analysis excluding volume outlier

units and using restricted cubic splines to model volume was also performed.

RESULTS: After adjusting for confounding, there was a significant relationship

between annual volume and ultimate acute hospital mortality (p < 0.02). The first

interaction test revealed a strong interaction between annual volume and

admission type, with a more pronounced volume-outcome relationship for

non-surgical admissions (p < 0.001). The second interaction test between annual

volume and initial acute severity of respiratory failure was not statistically

significant (p = 0.12). The analysis using restricted cubic splines demonstrated

a similar graphical relationship but the results were not statistically

significant (p = 0.87). CONCLUSIONS: A volume-outcome relationship was

demonstrated for mechanically ventilated admissions to adult, general critical

care units in the UK. The relationship is sensitive to the modelling approach

used.

FAU - Shahin, Jason

AU - Shahin J

AD - Intensive Care National Audit and Research Centre, London, UK,

jason.shahin@mcgill.ca.

FAU - Harrison, D A

AU - Harrison DA

FAU - Rowan, K M

AU - Rowan KM

LA - eng

PT - Journal Article

PT - Research Support, Non-U.S. Gov't

DEP - 20140207

PL - United States

TA - Intensive Care Med

JT - Intensive care medicine

JID - 7704851

SB - IM

CIN - Intensive Care Med. 2014 Mar;40(3):453-5. PMID: 24504642

MH - Adult

MH - Critical Illness/mortality

MH - Databases, Factual

MH - Diagnosis-Related Groups

MH - Female

MH - *Hospital Mortality

MH - Humans

MH - Intensive Care Units/*statistics & numerical data

MH - Length of Stay/statistics & numerical data

MH - Male

MH - Middle Aged

MH - Multivariate Analysis

MH - Outcome and Process Assessment, Health Care

MH - Patient Admission/*statistics & numerical data

MH - Respiration, Artificial/*mortality/statistics & numerical data

MH - Respiratory Insufficiency/*mortality

MH - Retrospective Studies

MH - United Kingdom

EDAT- 2014/02/08 06:00

MHDA- 2018/01/18 06:00

CRDT- 2014/02/08 06:00

PHST- 2013/11/20 00:00 [received]

PHST- 2013/12/20 00:00 [accepted]

PHST- 2014/02/08 06:00 [entrez]

PHST- 2014/02/08 06:00 [pubmed]

PHST- 2018/01/18 06:00 [medline]

AID - 10.1007/s00134-013-3205-4 [doi]

PST - ppublish

SO - Intensive Care Med. 2014 Mar;40(3):353-60. doi: 10.1007/s00134-013-3205-4. Epub

2014 Feb 7.

**Annexe 2- Articles sélectionnés comme pertinents compte tenu de la question PICO**

1. **PMID- 25148726**

OWN - NLM

STAT- MEDLINE

DCOM- 20150916

LR - 20220318

IS - 1466-609X (Electronic)

IS - 1364-8535 (Print)

IS - 1364-8535 (Linking)

VI - 18

IP - 4

DP - 2014 Aug 23

TI - Determining delayed admission to intensive care unit for mechanically ventilated

patients in the emergency department.

PG - 485

LID - 10.1186/s13054-014-0485-1 [doi]

LID - 485

AB - INTRODUCTION: The adverse effects of delayed admission to the intensive care unit

(ICU) have been recognized in previous studies. However, the definitions of

delayed admission varies across studies. This study proposed a model to define

"delayed admission", and explored the effect of ICU-waiting time on patients'

outcome. METHODS: This retrospective cohort study included non-traumatic adult

patients on mechanical ventilation in the emergency department (ED), from July

2009 to June 2010. The primary outcomes measures were 21-ventilator-day mortality

and prolonged hospital stays (over 30 days). Models of Cox regression and

logistic regression were used for multivariate analysis. The non-delayed

ICU-waiting was defined as a period in which the time effect on mortality was not

statistically significant in a Cox regression model. To identify a suitable

cut-off point between "delayed" and "non-delayed", subsets from the overall data

were made based on ICU-waiting time and the hazard ratio of ICU-waiting hour in

each subset was iteratively calculated. The cut-off time was then used to

evaluate the impact of delayed ICU admission on mortality and prolonged length of

hospital stay. RESULTS: The final analysis included 1,242 patients. The time

effect on mortality emerged after 4 hours, thus we deduced ICU-waiting time in

ED > 4 hours as delayed. By logistic regression analysis, delayed ICU admission

affected the outcomes of 21 ventilator-days mortality and prolonged hospital

stay, with odds ratio of 1.41 (95% confidence interval, 1.05 to 1.89) and 1.56

(95% confidence interval, 1.07 to 2.27) respectively. CONCLUSIONS: For patients

on mechanical ventilation at the ED, delayed ICU admission is associated with

higher probability of mortality and additional resource expenditure. A benchmark

waiting time of no more than 4 hours for ICU admission is recommended.

FAU - Hung, Shih-Chiang

AU - Hung SC

FAU - Kung, Chia-Te

AU - Kung CT

FAU - Hung, Chih-Wei

AU - Hung CW

FAU - Liu, Ber-Ming

AU - Liu BM

FAU - Liu, Jien-Wei

AU - Liu JW

FAU - Chew, Ghee

AU - Chew G

FAU - Chuang, Hung-Yi

AU - Chuang HY

FAU - Lee, Wen-Huei

AU - Lee WH

FAU - Lee, Tzu-Chi

AU - Lee TC

LA - eng

PT - Journal Article

DEP - 20140823

PL - England

TA - Crit Care

JT - Critical care (London, England)

JID - 9801902

SB - IM

MH - APACHE

MH - Aged

MH - Confidence Intervals

MH - Critical Care

MH - Diagnosis-Related Groups

MH - Emergency Service, Hospital/*organization & administration/statistics & numerical

data

MH - Female

MH - Glasgow Coma Scale

MH - Hospital Bed Capacity

MH - *Hospital Mortality

MH - Humans

MH - Intensive Care Units/economics/organization & administration/*statistics &

numerical data

MH - Length of Stay/economics/*statistics & numerical data

MH - Logistic Models

MH - Male

MH - Odds Ratio

MH - Patient Admission/economics/*statistics & numerical data

MH - Proportional Hazards Models

MH - Respiration, Artificial/*mortality/standards

MH - Respiratory Insufficiency/etiology/*mortality/therapy

MH - Retrospective Studies

MH - Taiwan/epidemiology

MH - Time Factors

MH - Triage/organization & administration/standards

PMC - PMC4175615

EDAT- 2014/08/26 06:00

MHDA- 2015/09/17 06:00

CRDT- 2014/08/24 06:00

PHST- 2014/03/29 00:00 [received]

PHST- 2014/07/29 00:00 [accepted]

PHST- 2014/08/24 06:00 [entrez]

PHST- 2014/08/26 06:00 [pubmed]

PHST- 2015/09/17 06:00 [medline]

AID - s13054-014-0485-1 [pii]

AID - 485 [pii]

AID - 10.1186/s13054-014-0485-1 [doi]

PST - epublish

SO - Crit Care. 2014 Aug 23;18(4):485. doi: 10.1186/s13054-014-0485-1.

1. **PMID- 27742520**

OWN - NLM

STAT- MEDLINE

DCOM- 20170320

LR - 20230725

IS - 1532-8171 (Electronic)

IS - 0735-6757 (Linking)

VI - 35

IP - 1

DP - 2017 Jan

TI - Impact of delayed admission to intensive care units on patients with acute

respiratory failure.

PG - 39-44

LID - S0735-6757(16)30677-5 [pii]

LID - 10.1016/j.ajem.2016.09.066 [doi]

AB - BACKGROUND/PURPOSE: To determine the impact of delayed admission to the intensive

care unit (ICU) on the clinical outcomes of patients with acute respiratory

failure (ARF) in the emergency department (ED). METHODS: This retrospective

cohort study included non-traumatic adult patients with ARF and mechanical

ventilation support in the ED of a tertiary university hospital in Taiwan from

January 1, 2013, to August 31, 2013. Clinical data were extracted from chart

records. The primary and secondary outcome measures were a prolonged hospital

stay (>30 days) and the in-hospital crude mortality within 90 days, respectively.

RESULTS: For 267 eligible patients (age range 21.0-98.0 years, mean 70.5±15.1

years; male 184, 68.9%), multivariate analysis was used to determine the

significant adverse effects of an ED stay >1.0 hour on in-hospital crude

mortality (odds ratio 2.19, P<.05), which was thus defined as delayed ICU

admission. In-hospital mortality significantly differed between patients with

delayed ICU admission and those without delayed admission, as revealed by the

Kaplan-Meier survival curves (P<.05). Moreover, a linear-by-linear correlation

was observed between the length of ICU waiting time in the ED and the lengths of

total hospital stay (r=0.152, P<.05), ICU stay (r=0.148, P<.05), and ventilator

support (r=0.222, P<.05). CONCLUSIONS: For patients with ARF who required

mechanical ventilation support and intensive care, a delayed ICU admission more

than 1.0 hour is a strong determinant of mortality and is associated with a

longer ICU stay and a longer need for ventilation.

CI - Copyright © 2016 Elsevier Inc. All rights reserved.

FAU - Hsieh, Chih-Chia

AU - Hsieh CC

AD - Department of Emergency Medicine, National Cheng Kung University Hospital,

College of Medicine, National Cheng Kung University, Tainan, Taiwan.

FAU - Lee, Ching-Chi

AU - Lee CC

AD - Department of Internal Medicine, Madou Sin-Lau Hospital, Tainan, Taiwan; Graduate

Institute of Medical Sciences, College of Health Sciences, Chang Jung Christian

University, Tainan, Taiwan.

FAU - Hsu, Hsiang-Chin

AU - Hsu HC

AD - Department of Emergency Medicine, National Cheng Kung University Hospital,

College of Medicine, National Cheng Kung University, Tainan, Taiwan.

FAU - Shih, Hsin-I

AU - Shih HI

AD - Department of Emergency Medicine, National Cheng Kung University Hospital,

College of Medicine, National Cheng Kung University, Tainan, Taiwan; Department

of Public Health, National Cheng Kung University Hospital, College of Medicine,

National Cheng Kung University, Tainan, Taiwan.

FAU - Lu, Chien-Hsin

AU - Lu CH

AD - Department of Emergency Medicine, National Cheng Kung University Hospital,

College of Medicine, National Cheng Kung University, Tainan, Taiwan.

FAU - Lin, Chih-Hao

AU - Lin CH

AD - Department of Emergency Medicine, National Cheng Kung University Hospital,

College of Medicine, National Cheng Kung University, Tainan, Taiwan. Electronic

address: emergency.lin@gmail.com.

LA - eng

PT - Journal Article

DEP - 20160930

PL - United States

TA - Am J Emerg Med

JT - The American journal of emergency medicine

JID - 8309942

SB - IM

CIN - Am J Emerg Med. 2017 Jun;35(6):914-915. PMID: 28318801

CIN - Am J Emerg Med. 2017 Oct;35(10):1571-1572. PMID: 28502761

CIN - Emerg Med Australas. 2023 Aug;35(4):612-617. PMID: 36718053

MH - Acute Disease

MH - Adult

MH - Aged

MH - Aged, 80 and over

MH - Cohort Studies

MH - Emergency Service, Hospital

MH - Female

MH - *Hospital Mortality

MH - *Hospitalization

MH - Humans

MH - *Intensive Care Units

MH - Length of Stay/*statistics & numerical data

MH - Male

MH - Middle Aged

MH - Multivariate Analysis

MH - Odds Ratio

MH - Respiration, Artificial

MH - Respiratory Insufficiency/mortality/*therapy

MH - Retrospective Studies

MH - Time Factors

MH - Young Adult

EDAT- 2016/10/16 06:00

MHDA- 2017/03/21 06:00

CRDT- 2016/10/16 06:00

PHST- 2016/05/27 00:00 [received]

PHST- 2016/08/31 00:00 [revised]

PHST- 2016/09/29 00:00 [accepted]

PHST- 2016/10/16 06:00 [pubmed]

PHST- 2017/03/21 06:00 [medline]

PHST- 2016/10/16 06:00 [entrez]

AID - S0735-6757(16)30677-5 [pii]

AID - 10.1016/j.ajem.2016.09.066 [doi]

PST - ppublish

SO - Am J Emerg Med. 2017 Jan;35(1):39-44. doi: 10.1016/j.ajem.2016.09.066. Epub 2016

Sep 30.

1. **PMID- 31575708**

OWN - NLM

STAT- MEDLINE

DCOM- 20201125

LR - 20201125

IS - 1943-3654 (Electronic)

IS - 0020-1324 (Print)

IS - 0020-1324 (Linking)

VI - 65

IP - 1

DP - 2020 Jan

TI - Noninvasive Ventilation for Critically Ill Subjects With Acute Respiratory

Failure in the Emergency Department.

PG - 82-90

LID - 10.4187/respcare.07111 [doi]

AB - BACKGROUND: We aimed to investigate the association between noninvasive

ventilation (NIV) initiated in the emergency department and patient outcomes for

those requiring invasive mechanical ventilation so that we could understand the

effect of extended NIV use (ie, > 4 h) prior to invasive mechanical ventilation

on patient outcomes. METHODS: We conducted a retrospective single-center cohort

study at an academic tertiary care hospital center. All emergency department

patients with acute respiratory failure requiring invasive mechanical ventilation

and admission to the ICU within 48 h of initial presentation over a 24-month

period were included. RESULTS: Subject characteristics, ventilator parameters,

and clinical course were captured via electronic query, respiratory billing data,

and standardized chart abstraction. A total of 431 subjects with acute

respiratory failure requiring invasive mechanical ventilation within 48 h of

arrival were identified, of whom 115 (26.7%) were exposed to NIV prior to

invasive mechanical ventilation, with a median duration of 4 h (interquartile

range 1.9-9.3). Based on a multivariable model controlling for covariates, any

NIV exposure prior to invasive mechanical ventilation was not associated with an

increased odds of persistent organ dysfunction or death. However, in the subset

of subjects exposed to NIV, extended NIV use (ie, > 4 h) prior to invasive

mechanical ventilation was associated with increased odds of persistent organ

dysfunction or death (odds ratio 4.11, 95% CI 1.51-11.19). Extended NIV use was

also associated with increased odds of in-hospital mortality (odds ratio 4.02,

95% CI 1.51-10.74). CONCLUSIONS: Although any exposure to NIV prior to invasive

mechanical ventilation did not appear to affect morbidity and mortality, extended

NIV use prior to invasive mechanical ventilation was associated with worse

patient outcomes, suggesting a need for additional study to better understand the

ramifications of duration of NIV use prior to failure on outcomes. Given this

early timeframe for intervention, future studies should be collaborations between

the emergency department and ICU.

CI - Copyright © 2020 by Daedalus Enterprises.

FAU - Goel, Neha N

AU - Goel NN

AD - Division of Pulmonary, Critical Care, and Sleep Medicine, Department of Medicine,

Icahn School of Medicine at Mount Sinai, New York, New York.

neha.goel@mountsinai.org.

FAU - Owyang, Clark

AU - Owyang C

AD - Department of Emergency Medicine, Icahn School of Medicine at Mount Sinai, New

York, New York.

FAU - Ranginwala, Shamsuddoha

AU - Ranginwala S

AD - Department of Respiratory Therapy, Icahn School of Medicine at Mount Sinai, New

York, New York.

FAU - Loo, George T

AU - Loo GT

AD - Department of Emergency Medicine, Icahn School of Medicine at Mount Sinai, New

York, New York.

AD - Department of Population Health Science and Policy, Icahn School of Medicine at

Mount Sinai, New York, New York.

FAU - Richardson, Lynne D

AU - Richardson LD

AD - Department of Emergency Medicine, Icahn School of Medicine at Mount Sinai, New

York, New York.

AD - Department of Population Health Science and Policy, Icahn School of Medicine at

Mount Sinai, New York, New York.

FAU - Mathews, Kusum S

AU - Mathews KS

AD - Division of Pulmonary, Critical Care, and Sleep Medicine, Department of Medicine,

Icahn School of Medicine at Mount Sinai, New York, New York.

LA - eng

GR - K23 HL130648/HL/NHLBI NIH HHS/United States

PT - Journal Article

DEP - 20191001

PL - United States

TA - Respir Care

JT - Respiratory care

JID - 7510357

SB - IM

MH - Cohort Studies

MH - Critical Illness

MH - Emergency Service, Hospital

MH - Hospital Mortality

MH - Humans

MH - Intensive Care Units

MH - Intubation/*mortality

MH - Noninvasive Ventilation/*mortality

MH - Respiration, Artificial/*mortality

MH - Respiratory Insufficiency/*therapy

MH - Retrospective Studies

MH - Treatment Failure

PMC - PMC7119184

OTO - NOTNLM

OT - bi-level

OT - critically ill

OT - emergency department

OT - mechanical ventilation

OT - noninvasive ventilation

OT - respiratory failure

COIS- Dr Goel presented a version of this paper was presented at the American Thoracic

Society 2018 International Conference, held May 18-23, 2018, in San Diego,

California. Dr Goel is partially supported by National Institutes of Health (NIH)

National Heart, Lung, and Blood Institute Award DHHS – 1T32 HL129974-PI:

Richardson. Dr Mathews is partially supported by NIH National Heart, Lung, and

Blood Institute Award 1K23HL130648-PI: Mathews. This work was supported in part

through the Mount Sinai Data Warehouse resources and staff expertise provided by

Scientific Computing at the Icahn School of Medicine at Mount Sinai. The other

authors have disclosed no conflicts of interest.

EDAT- 2019/10/03 06:00

MHDA- 2020/11/26 06:00

CRDT- 2019/10/03 06:00

PHST- 2019/10/03 06:00 [pubmed]

PHST- 2020/11/26 06:00 [medline]

PHST- 2019/10/03 06:00 [entrez]

AID - respcare.07111 [pii]

AID - RC-07111 [pii]

AID - 10.4187/respcare.07111 [doi]

PST - ppublish

SO - Respir Care. 2020 Jan;65(1):82-90. doi: 10.4187/respcare.07111. Epub 2019 Oct 1.

1. **PMID- 35895629**

OWN - NLM

STAT- MEDLINE

DCOM- 20230303

LR - 20230310

IS - 2325-6621 (Electronic)

IS - 2329-6933 (Print)

IS - 2325-6621 (Linking)

VI - 20

IP - 3

DP - 2023 Mar

TI - Among-Hospital Variation in Intensive Care Unit Admission Practices and

Associated Outcomes for Patients with Acute Respiratory Failure.

PG - 406-413

LID - 10.1513/AnnalsATS.202205-429OC [doi]

AB - Rationale: We have previously shown that hospital strain is associated with

intensive care unit (ICU) admission and that ICU admission, compared with ward

admission, may benefit certain patients with acute respiratory failure (ARF).

Objectives: To understand how strain-process-outcomes relationships in patients

with ARF may vary among hospitals and what hospital practice differences may

account for such variation. Methods: We examined high-acuity patients with ARF

who did not require mechanical ventilation or vasopressors in the emergency

department (ED) and were admitted to 27 U.S. hospitals from 2013 to 2018.

Stratifying by hospital, we compared hospital strain-ICU admission relationships

and hospital length of stay (LOS) and mortality among patients initially admitted

to the ICU versus the ward using hospital strain as a previously validated

instrumental variable. We also surveyed hospital practices and, in exploratory

analyses, evaluated their associations with the above processes and outcomes.

Results: There was significant among-hospital variation in ICU admission rates,

in hospital strain-ICU admission relationships, and in the association of ICU

admission with hospital LOS and hospital mortality. Overall, ED patients with ARF

(n = 45,339) experienced a 0.82-day shorter median hospital LOS if admitted

initially to the ICU compared with the ward, but among the 27 hospitals

(n = 224-3,324), this effect varied from 5.85 days shorter (95% confidence

interval [CI], -8.84 to -2.86; P < 0.001) to 4.38 days longer (95% CI, 1.86-6.90;

P = 0.001). Corresponding ranges for in-hospital mortality with ICU compared with

ward admission revealed odds ratios from 0.08 (95% CI, 0.01-0.56; P < 0.007) to

8.89 (95% CI, 1.60-79.85; P = 0.016) among patients with ARF (pooled odds ratio,

0.75). In exploratory analyses, only a small number of measured hospital

practices-the presence of a sepsis ED disposition guideline and maximum ED

patient capacity-were potentially associated with hospital strain-ICU admission

relationships. Conclusions: Hospitals vary considerably in ICU admission rates,

the sensitivity of those rates to hospital capacity strain, and the benefits of

ICU admission for patients with ARF not requiring life support therapies in the

ED. Future work is needed to more fully identify hospital-level factors

contributing to these relationships.

FAU - Anesi, George L

AU - Anesi GL

AUID- ORCID: 0000-0003-4585-0714

AD - Division of Pulmonary, Allergy, and Critical Care, Perelman School of Medicine.

AD - Leonard Davis Institute of Health Economics.

AD - Palliative and Advanced Illness Research Center, Perelman School of Medicine.

FAU - Dress, Erich

AU - Dress E

AD - Palliative and Advanced Illness Research Center, Perelman School of Medicine.

FAU - Chowdhury, Marzana

AU - Chowdhury M

AD - Palliative and Advanced Illness Research Center, Perelman School of Medicine.

FAU - Wang, Wei

AU - Wang W

AD - Palliative and Advanced Illness Research Center, Perelman School of Medicine.

FAU - Small, Dylan S

AU - Small DS

AD - Department of Statistics, The Wharton School.

FAU - Delgado, M Kit

AU - Delgado MK

AD - Leonard Davis Institute of Health Economics.

AD - Palliative and Advanced Illness Research Center, Perelman School of Medicine.

AD - Center for Emergency Care Policy and Research, Department of Emergency Medicine,

Perelman School of Medicine, and.

FAU - Bayes, Brian

AU - Bayes B

AD - Palliative and Advanced Illness Research Center, Perelman School of Medicine.

FAU - Szymczak, Julia E

AU - Szymczak JE

AD - Department of Biostatistics, Epidemiology, and Informatics, Perelman School of

Medicine, University of Pennsylvania, Philadelphia, Pennsylvania; and.

FAU - Glassman, Lindsay W

AU - Glassman LW

AD - Department of Biostatistics, Epidemiology, and Informatics, Perelman School of

Medicine, University of Pennsylvania, Philadelphia, Pennsylvania; and.

FAU - Barreda, Fernando X

AU - Barreda FX

AD - Division of Research, Kaiser Permanente, Oakland, California.

FAU - Weiner, Jonathan Z

AU - Weiner JZ

AD - Division of Research, Kaiser Permanente, Oakland, California.

FAU - Escobar, Gabriel J

AU - Escobar GJ

AD - Division of Research, Kaiser Permanente, Oakland, California.

FAU - Halpern, Scott D

AU - Halpern SD

AUID- ORCID: 0000-0002-3603-4769

AD - Division of Pulmonary, Allergy, and Critical Care, Perelman School of Medicine.

AD - Leonard Davis Institute of Health Economics.

AD - Palliative and Advanced Illness Research Center, Perelman School of Medicine.

AD - Department of Biostatistics, Epidemiology, and Informatics, Perelman School of

Medicine, University of Pennsylvania, Philadelphia, Pennsylvania; and.

FAU - Liu, Vincent X

AU - Liu VX

AD - Division of Research, Kaiser Permanente, Oakland, California.

LA - eng

GR - K23 HL161353/HL/NHLBI NIH HHS/United States

GR - L30 HL138827/HL/NHLBI NIH HHS/United States

GR - L30 HL154200/HL/NHLBI NIH HHS/United States

GR - R01 HL136719/HL/NHLBI NIH HHS/United States

PT - Journal Article

PT - Research Support, N.I.H., Extramural

PL - United States

TA - Ann Am Thorac Soc

JT - Annals of the American Thoracic Society

JID - 101600811

SB - IM

CIN - Ann Am Thorac Soc. 2023 Mar;20(3):364-366. PMID: 36856718

MH - Humans

MH - Hospitalization

MH - Length of Stay

MH - Intensive Care Units

MH - Emergency Service, Hospital

MH - Hospitals

MH - Hospital Mortality

MH - *Respiratory Distress Syndrome

MH - *Respiratory Insufficiency/therapy

MH - Retrospective Studies

PMC - PMC9993147

OTO - NOTNLM

OT - acute respiratory failure

OT - hospital strain

OT - hospital variation

OT - intensive care unit

OT - processes of care

EDAT- 2022/07/28 06:00

MHDA- 2023/03/04 06:00

PMCR- 2024/03/01

CRDT- 2022/07/27 13:34

PHST- 2024/03/01 00:00 [pmc-release]

PHST- 2022/07/28 06:00 [pubmed]

PHST- 2023/03/04 06:00 [medline]

PHST- 2022/07/27 13:34 [entrez]

AID - 10.1513/AnnalsATS.202205-429OC [doi]

PST - ppublish

SO - Ann Am Thorac Soc. 2023 Mar;20(3):406-413. doi: 10.1513/AnnalsATS.202205-429OC.

1. **PMID- 26210474**

OWN - NLM

STAT- MEDLINE

DCOM- 20160711

LR - 20181202

IS - 1432-1750 (Electronic)

IS - 0341-2040 (Linking)

VI - 193

IP - 5

DP - 2015 Oct

TI - Where is Noninvasive Ventilation Actually Delivered for Acute Respiratory

Failure?

PG - 779-88

LID - 10.1007/s00408-015-9766-y [doi]

AB - PURPOSE: Few studies have examined locations of noninvasive ventilation (NIV)

application for acute respiratory failure (ARF). We aimed to track actual

locations of NIV delivery and related outcomes. METHODS: Observational cohort

study based at 8 acute care hospitals in Massachusetts on adult patients admitted

for ARF requiring ventilatory support during pre-determined time intervals.

RESULTS: Of 1225 ventilator starts, 499 were NIV; 209 (42%) in intensive care

units (ICU), 185 (37%) in emergency departments (ED), 91 (18%) on general wards,

and 14 (3%) in other units. Utilization (% of all ventilator starts) (1), success

(2) and in-hospital mortality (3) rates for patients initiated on NIV in ICU, ED,

and general and other wards were (1) 38, 36, 73, and 52%, (2) 60, 77, 68, and 93%

and (3) 25, 12, 17, and 0%, respectively (p < 0.05 for all). Patients with

acute-on-chronic lung disease (ACLD) and acute pulmonary edema (APE) were begun

on NIV most often in EDs and patients with 'de novo' ARF and neurologic disorders

most often in ICU's. Approximately 2/3 of patients begun on NIV outside of ICUs

were transferred within 72 h to ICUs, wards or other units. CONCLUSIONS: Most NIV

starts occurred in ICUs and EDs but utilization rate was highest (>50%) on

general wards where a fifth of NIV starts took place. Actual location depended on

etiology of ARF as patients with ACLD and APE were started more often in EDs and

"de novo" ARF in ICU. NIV failure and mortality rates were higher in ICUs related

to the greater proportion of patients with "de novo" ARF.

FAU - Ozsancak Ugurlu, Aylin

AU - Ozsancak Ugurlu A

AD - Department of Pulmonary Disease, Baskent University, Oymaci sok. No: 2, 34662,

Altunizade/Istanbul, Turkey. aozsancak@hotmail.com.

FAU - Sidhom, Samy S

AU - Sidhom SS

AD - Pulmonary Department, Newton-Wellesley Hospital, Newton, MA, USA.

FAU - Khodabandeh, Ali

AU - Khodabandeh A

AD - Mass Lung and Allergy PC, Leominster, MA, USA.

FAU - Ieong, Michael

AU - Ieong M

AD - Boston Medical Center, Boston, MA, USA.

FAU - Mohr, Chester

AU - Mohr C

AD - Cape Cod Health Systems, Hyannis, MA, USA.

FAU - Lin, Denis Y

AU - Lin DY

AD - Lowell General Hospital, Lowell, MA, USA.

FAU - Buchwald, Irwin

AU - Buchwald I

AD - Saints Medical Center, Lowell, MA, USA.

FAU - Bahhady, Imad

AU - Bahhady I

AD - Morton Hospital, Taunton, MA, USA.

FAU - Wengryn, John

AU - Wengryn J

AD - Jordan Hospital, Plymouth, MA, USA.

FAU - Maheshwari, Vinay

AU - Maheshwari V

AD - Pulmonary Associates, Newark, DE, USA.

FAU - Hill, Nicholas S

AU - Hill NS

AD - Department of Pulmonary, Critical Care and Sleep Medicine, Tufts Medical Center,

Boston, MA, USA.

LA - eng

SI - ClinicalTrials.gov/NCT00458926

PT - Journal Article

PT - Observational Study

PT - Research Support, Non-U.S. Gov't

DEP - 20150726

PL - United States

TA - Lung

JT - Lung

JID - 7701875

SB - IM

MH - Acute Disease

MH - Aged

MH - Aged, 80 and over

MH - Chronic Disease

MH - Emergency Service, Hospital/*statistics & numerical data

MH - Female

MH - Hospital Mortality

MH - Hospitals

MH - Humans

MH - Intensive Care Units/*statistics & numerical data

MH - Male

MH - Massachusetts

MH - Middle Aged

MH - Nervous System Diseases/complications

MH - Noninvasive Ventilation/*statistics & numerical data

MH - Patients' Rooms/*statistics & numerical data

MH - Pulmonary Edema/complications/therapy

MH - Respiratory Insufficiency/etiology/*therapy

MH - Treatment Outcome

OTO - NOTNLM

OT - Acute respiratory failure

OT - Emergency care

OT - General wards

OT - Intensive care units

OT - Noninvasive ventilation

EDAT- 2015/07/27 06:00

MHDA- 2016/07/12 06:00

CRDT- 2015/07/27 06:00

PHST- 2015/01/29 00:00 [received]

PHST- 2015/07/19 00:00 [accepted]

PHST- 2015/07/27 06:00 [entrez]

PHST- 2015/07/27 06:00 [pubmed]

PHST- 2016/07/12 06:00 [medline]

AID - 10.1007/s00408-015-9766-y [pii]

AID - 10.1007/s00408-015-9766-y [doi]

PST - ppublish

SO - Lung. 2015 Oct;193(5):779-88. doi: 10.1007/s00408-015-9766-y. Epub 2015 Jul 26.

1. **PMID- 23185988**

OWN - NLM

STAT- MEDLINE

DCOM- 20140224

LR - 20190116

IS - 1029-2403 (Electronic)

IS - 1026-8022 (Linking)

VI - 54

IP - 8

DP - 2013 Aug

TI - Delayed intensive care unit admission is associated with increased mortality in

patients with cancer with acute respiratory failure.

PG - 1724-9

LID - 10.3109/10428194.2012.753446 [doi]

AB - Acute respiratory failure (ARF) is the leading reason for intensive care unit

(ICU) admission in patients with cancer. The aim of this study was to identify

early predictors of death in patients with cancer admitted to the ICU for ARF who

were not intubated at admission. We conducted analysis of a prospective

randomized controlled trial including 219 patients with cancer with ARF in which

day-28 mortality was a secondary endpoint. Mortality at day 28 was 31.1%. By

multivariate analysis, independent predictors of day-28 mortality were: age (odds

ratio [OR] 1.30/10 years, 95% confidence interval [CI] [1.01-1.68], p = 0.04),

more than one line of chemotherapy (OR 2.14, 95% CI [1.08-4.21], p = 0.03), time

between respiratory symptoms onset and ICU admission > 2 days (OR 2.50, 95% CI

[1.25-5.02], p = 0.01), oxygen flow at admission (OR 1.07/L, 95% CI [1.00-1.14],

p = 0.04) and extra-respiratory symptoms (OR 2.84, 95%CI [1.30-6.21], p = 0.01).

After adjustment for the logistic organ dysfunction (LOD) score at admission,

only time between respiratory symptoms onset and ICU admission > 2 days and LOD

score were independently associated with day-28 mortality. Determinants of death

include both factors non-amenable to change, and delay in ARF management. These

results suggest that early intensive care management of patients with cancer with

ARF may translate to better survival.

FAU - Mokart, Djamel

AU - Mokart D

AD - Réanimation Polyvalente, Institut Paoli-Calmettes, Marseille, France.

mokartd@ipc.unicancer.fr

FAU - Lambert, Jérôme

AU - Lambert J

FAU - Schnell, David

AU - Schnell D

FAU - Fouché, Louis

AU - Fouché L

FAU - Rabbat, Antoine

AU - Rabbat A

FAU - Kouatchet, Achille

AU - Kouatchet A

FAU - Lemiale, Virginie

AU - Lemiale V

FAU - Vincent, François

AU - Vincent F

FAU - Lengliné, Etienne

AU - Lengliné E

FAU - Bruneel, Fabrice

AU - Bruneel F

FAU - Pene, Frederic

AU - Pene F

FAU - Chevret, Sylvie

AU - Chevret S

FAU - Azoulay, Elie

AU - Azoulay E

LA - eng

PT - Journal Article

DEP - 20121226

PL - United States

TA - Leuk Lymphoma

JT - Leukemia & lymphoma

JID - 9007422

SB - IM

MH - Aged

MH - Female

MH - Hospital Mortality

MH - Humans

MH - *Intensive Care Units

MH - Male

MH - Middle Aged

MH - Neoplasms/*complications/*mortality

MH - *Patient Admission

MH - Prognosis

MH - Prospective Studies

MH - Respiratory Insufficiency/*complications/*mortality

EDAT- 2012/11/29 06:00

MHDA- 2014/02/25 06:00

CRDT- 2012/11/29 06:00

PHST- 2012/11/29 06:00 [entrez]

PHST- 2012/11/29 06:00 [pubmed]

PHST- 2014/02/25 06:00 [medline]

AID - 10.3109/10428194.2012.753446 [doi]

PST - ppublish

SO - Leuk Lymphoma. 2013 Aug;54(8):1724-9. doi: 10.3109/10428194.2012.753446. Epub

2012 Dec 26.

1. **PMID- 32389397**

OWN - NLM

STAT- MEDLINE

DCOM- 20200824

LR - 20201210

IS - 1532-8171 (Electronic)

IS - 0735-6757 (Linking)

VI - 38

IP - 7

DP - 2020 Jul

TI - High-flow nasal cannula oxygen therapy in acute respiratory failure at Emergency

Departments: A systematic review.

PG - 1508-1514

LID - S0735-6757(20)30323-5 [pii]

LID - 10.1016/j.ajem.2020.04.091 [doi]

AB - OBJECTIVES: The use of high-flow oxygen therapy (HFOT) through nasal cannula for

the management of acute respiratory failure at the emergency department (ED) has

been only sparsely studied. We conducted a systematic review of

randomized-controlled and quasi-experimental studies comparing the early use of

HFOT versus conventional oxygen therapy (COT) in patients with acute respiratory

failure admitted to EDs. METHODS: A systematic research of literature was carried

out for all published control trials comparing HFOT with COT in adult patients

admitted in EDs. Eligible data were extracted from Medline, Embase, Pascal, Web

of Science and the Cochrane database. The primary outcome was the need for

mechanical ventilation, i.e. intubation or non-invasive ventilation as rescue

therapy. Secondary outcomes included respiratory rate, dyspnea level, ED length

of stay, intubation and mortality. RESULTS: Out of 1829 studies screened, five

studies including 673 patients were retained in the analysis (350 patients

treated with HFOT and 323 treated with COT). The need for mechanical ventilation

was similar in both treatments (RR = 0.75; 95% CI 0.41 to 1.35; P = 0.31;

I(2) = 16%). Respiratory rate was lower with HFOT (Mean difference (MD) = -3.14

breaths/min; 95% CI = -4.9 to -1.4; P < 0.001; I(2) = 39%), whereas sensation of

dyspnea did not differ. (MD = -1.04; 95% CI = -2.29 to -0.22; P = 0.08;

I(2) = 67%). ED length of stay and mortality were similar between groups.

CONCLUSION: The early use of HFOT in patients admitted to an ED for acute

respiratory failure did not reduce the need for mechanical ventilation as

compared to COT. However, HFOT decreased respiratory rate. REGISTRATION: PROSPERO

ID CRD42019125696.

CI - Copyright © 2020. Published by Elsevier Inc.

FAU - Marjanovic, Nicolas

AU - Marjanovic N

AD - CHU de Poitiers, Service des Urgences et SAMU 86, Poitiers, France; INSERM,

CIC-1402 Team 5 ALIVE, Poitiers, France; Université de Poitiers, Faculté de

Médecine et de Pharmacie de Poitiers, Poitiers, France. Electronic address:

marjanovic@chu-poitiers.fr.

FAU - Guénézan, Jérémy

AU - Guénézan J

AD - CHU de Poitiers, Service des Urgences et SAMU 86, Poitiers, France; Université de

Poitiers, Faculté de Médecine et de Pharmacie de Poitiers, Poitiers, France.

FAU - Frat, Jean-Pierre

AU - Frat JP

AD - INSERM, CIC-1402 Team 5 ALIVE, Poitiers, France; Université de Poitiers, Faculté

de Médecine et de Pharmacie de Poitiers, Poitiers, France; CHU de Poitiers,

Médecine Intensive Réanimation, Poitiers, France.

FAU - Mimoz, Olivier

AU - Mimoz O

AD - CHU de Poitiers, Service des Urgences et SAMU 86, Poitiers, France; Université de

Poitiers, Faculté de Médecine et de Pharmacie de Poitiers, Poitiers, France.

FAU - Thille, Arnaud W

AU - Thille AW

AD - INSERM, CIC-1402 Team 5 ALIVE, Poitiers, France; Université de Poitiers, Faculté

de Médecine et de Pharmacie de Poitiers, Poitiers, France; CHU de Poitiers,

Médecine Intensive Réanimation, Poitiers, France.

LA - eng

PT - Journal Article

PT - Systematic Review

DEP - 20200504

PL - United States

TA - Am J Emerg Med

JT - The American journal of emergency medicine

JID - 8309942

SB - IM

MH - Dyspnea/*therapy

MH - Emergency Service, Hospital

MH - Hospital Mortality

MH - Humans

MH - Length of Stay

MH - Oxygen Inhalation Therapy/*methods

MH - Respiration, Artificial/statistics & numerical data

MH - Respiratory Distress Syndrome/*therapy

MH - Respiratory Insufficiency/*therapy

MH - Respiratory Rate

OTO - NOTNLM

OT - Emergency department

OT - High-flow nasal cannula

OT - Respiratory failure

COIS- Declaration of competing interest NM reports travel expense coverage to attend

scientific meetings and payment for lectures from Fisher & Paykel. JPF reports

travel expense coverage to attend scientific meetings and personal fees from

Fisher & Paykel. AWT reports travel expense coverage to attend scientific

meetings and payment for lectures from Fisher & Paykel.

EDAT- 2020/05/12 06:00

MHDA- 2020/08/25 06:00

CRDT- 2020/05/12 06:00

PHST- 2020/01/26 00:00 [received]

PHST- 2020/04/17 00:00 [revised]

PHST- 2020/04/26 00:00 [accepted]

PHST- 2020/05/12 06:00 [pubmed]

PHST- 2020/08/25 06:00 [medline]

PHST- 2020/05/12 06:00 [entrez]

AID - S0735-6757(20)30323-5 [pii]

AID - 10.1016/j.ajem.2020.04.091 [doi]

PST - ppublish

SO - Am J Emerg Med. 2020 Jul;38(7):1508-1514. doi: 10.1016/j.ajem.2020.04.091. Epub

2020 May 4.

1. **PMID- 23650433**

OWN - NLM

STAT- MEDLINE

DCOM- 20140818

LR - 20191210

IS - 1943-3654 (Electronic)

IS - 0020-1324 (Linking)

VI - 58

IP - 12

DP - 2013 Dec

TI - Influence of the admission pattern on the outcome of patients admitted to a

respiratory intensive care unit: does a step-down admission differ from a step-up

one?

PG - 2053-60

LID - 10.4187/respcare.02225 [doi]

AB - BACKGROUND: The outcomes of patients admitted to a respiratory ICU (RICU) have

been evaluated in the past, but no study has considered the influence of location

prior to RICU admission. METHODS: We analyzed prospectively collected data from

326 consecutive patients admitted to a 7-bed RICU. The primary end points were

survival and severity of morbidity-related complications, evaluated according to

the patient's location prior to RICU admission. Three admission pathways were

considered: step-down for patients transferred from the ICUs of our hospital;

step-up for patients coming from our respiratory wards or other medical wards;

and directly for patients coming from the emergency department. The secondary end

point was the potential influence of several risk factors for morbidity and

mortality. RESULTS: Of the 326 subjects, 92 (28%) died. Overall, subjects

admitted in a step-up process had a significantly higher mortality (P < .001)

than subjects in the other groups. The mortality rate was 64% for subjects

admitted from respiratory ward, 43% for those from medical wards, and 18% for

subjects from both ICU and emergency department (respiratory ward vs medical ward

P = .04, respiratory ward vs emergency department P < .001, respiratory ward vs

ICU P < .001, medical ward vs emergency department P < .001, and medical ward vs

ICU P < .001). Subjects admitted from a respiratory ward had a lower albumin

level, and Simplified Acute Physiology Score II was significantly higher in

subjects following a step-up admission. About 30% of the subjects admitted from a

respiratory ward received noninvasive ventilation as a "ceiling treatment." The

highest odds ratios related to survival were subject location prior to RICU

admission and female sex. Lack of use of noninvasive ventilation, younger age,

female sex, higher albumin level, lower Simplified Acute Physiology Score II,

higher Barthel score, and absence of chronic heart failure were also

statistically associated with a lower risk of death. CONCLUSIONS: The pathway of

admission to a RICU is a determinant of outcome. Patients following a step-up

pattern are more likely to die. Other major determinants of survival are age,

nutritional status and female sex.

FAU - Valentini, Ilaria

AU - Valentini I

AD - Department of Specialist, Diagnostic and Experimental Medicine.

FAU - Pacilli, Angela Maria Grazia

AU - Pacilli AM

FAU - Carbonara, Paolo

AU - Carbonara P

FAU - Fasano, Luca

AU - Fasano L

FAU - Vitale, Rita

AU - Vitale R

FAU - Zenesini, Corrado

AU - Zenesini C

FAU - Melotti, Rita Maria

AU - Melotti RM

FAU - Faenza, Stefano

AU - Faenza S

FAU - Nava, Stefano

AU - Nava S

LA - eng

PT - Journal Article

DEP - 20130430

PL - United States

TA - Respir Care

JT - Respiratory care

JID - 7510357

SB - IM

MH - APACHE

MH - Adult

MH - Aged

MH - Aged, 80 and over

MH - Critical Pathways/standards

MH - *Emergency Medical Services/methods/statistics & numerical data

MH - Emergency Service, Hospital/*statistics & numerical data

MH - Female

MH - Humans

MH - Italy/epidemiology

MH - Male

MH - Middle Aged

MH - Organizational Policy

MH - Outcome and Process Assessment, Health Care

MH - *Patient Admission/standards/statistics & numerical data/trends

MH - Prospective Studies

MH - Respiratory Care Units/statistics & numerical data

MH - *Respiratory Insufficiency/etiology/mortality/therapy

MH - Risk Assessment

MH - Risk Factors

MH - Survival Analysis

MH - Transportation of Patients/*methods

OTO - NOTNLM

OT - ICU

OT - acute respiratory failure

OT - critically ill patient

OT - noninvasive ventilation

OT - respiratory ICU

OT - weaning

EDAT- 2013/05/08 06:00

MHDA- 2014/08/19 06:00

CRDT- 2013/05/08 06:00

PHST- 2013/05/08 06:00 [entrez]

PHST- 2013/05/08 06:00 [pubmed]

PHST- 2014/08/19 06:00 [medline]

AID - respcare.02225 [pii]

AID - 10.4187/respcare.02225 [doi]

PST - ppublish

SO - Respir Care. 2013 Dec;58(12):2053-60. doi: 10.4187/respcare.02225. Epub 2013 Apr

30.
