# Supplementary material for: Oxygen therapy in acute hypoxemic respiratory failure: guidelines from the SRLF-SFMU consensus conference
Source: Ann Intensive Care. 2024 Sep 5;14:140. doi: 10.1186/s13613-024-01367-2 (PMC11377397; doi:10.1186/s13613-024-01367-2)

**Appendix 3: HFNC** **vs. standard O_2_ according to disease:**INTUBATION in non-COVID-19 respiratory failure vs. COVID-19 respiratory failure


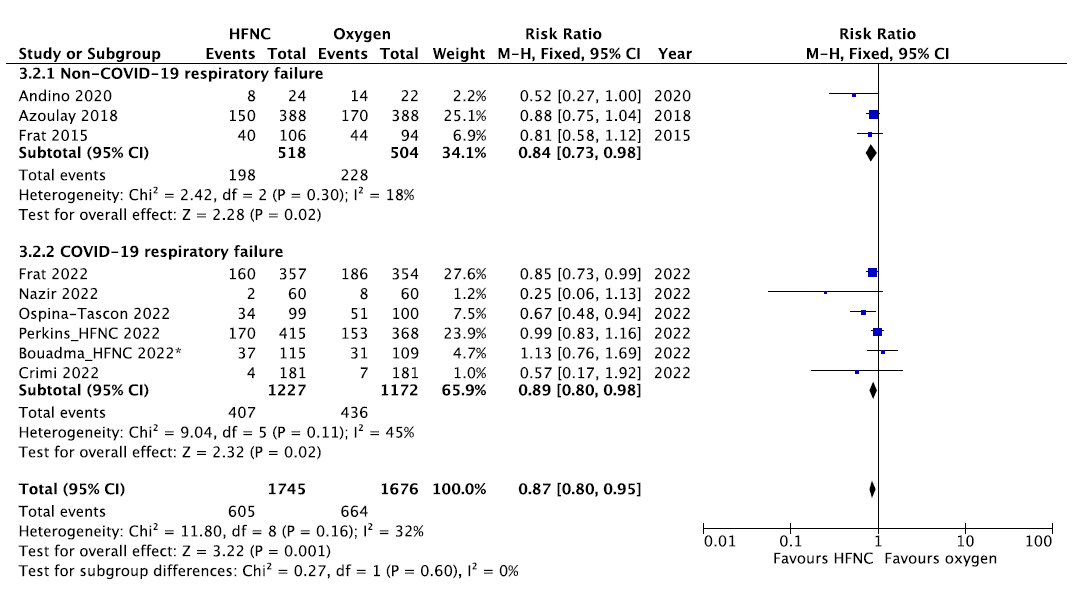

Supplement: Supplementary file 3 — Supplementary material 3. [file 13613_2024_1367_MOESM3_ESM.docx]
