# Supplementary figures and images for: Oxygen therapy in acute hypoxemic respiratory failure: guidelines from the SRLF-SFMU consensus conference
Source: Ann Intensive Care. 2024 Sep 5;14:140. doi: 10.1186/s13613-024-01367-2 (PMC11377397; doi:10.1186/s13613-024-01367-2)

## Slide 1
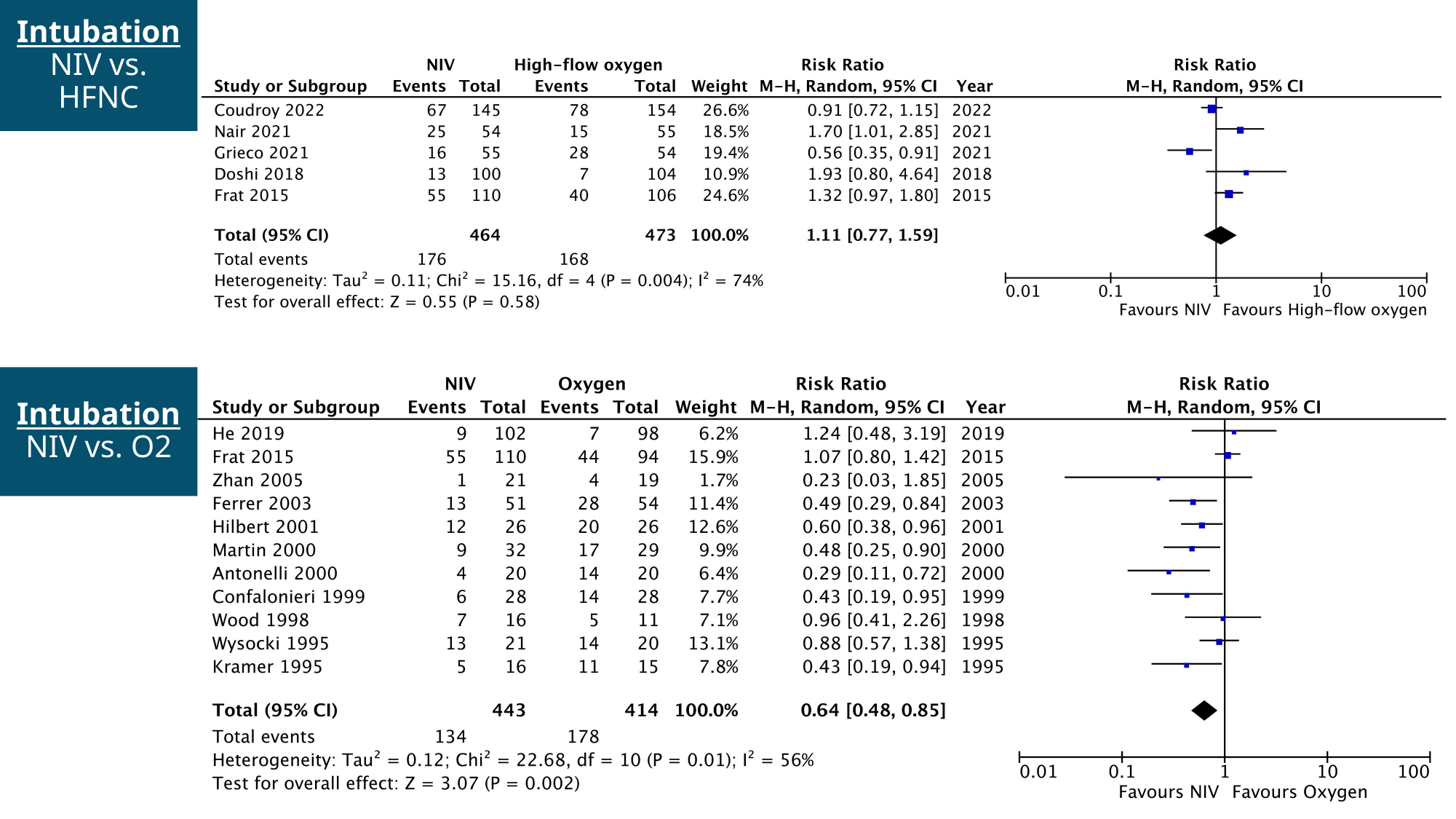

# Intubation NIV vs. HFNC
Intubation
NIV vs. O2

Supplement: Supplementary file 5 — Supplementary material 5. [file 13613_2024_1367_MOESM5_ESM.pptx]

## Slide 1
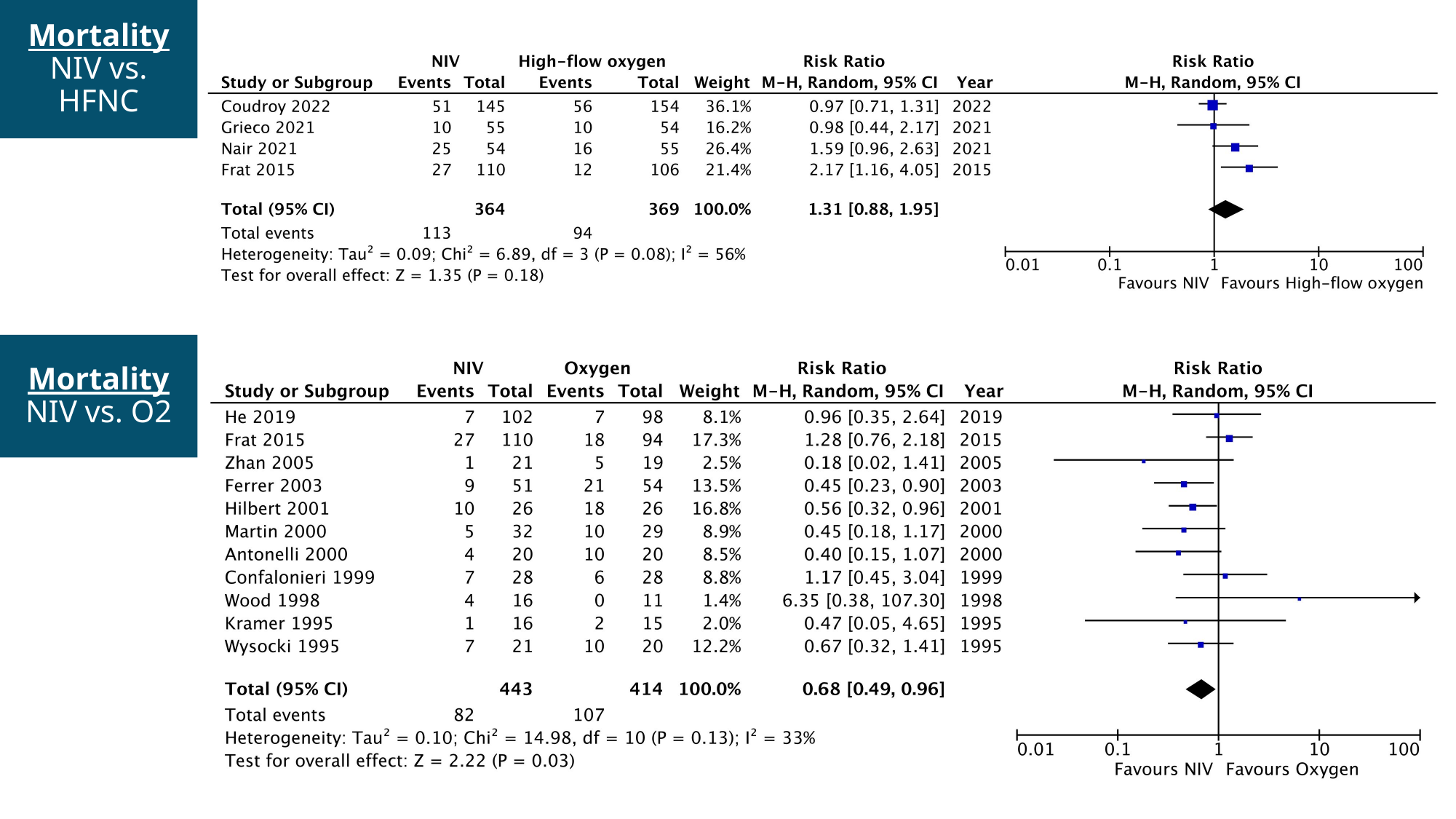

# Mortality NIV vs. HFNC
Mortality
NIV vs. O2

Supplement: Supplementary file 6 — Supplementary material 6. [file 13613_2024_1367_MOESM6_ESM.pptx]

## Slide 1
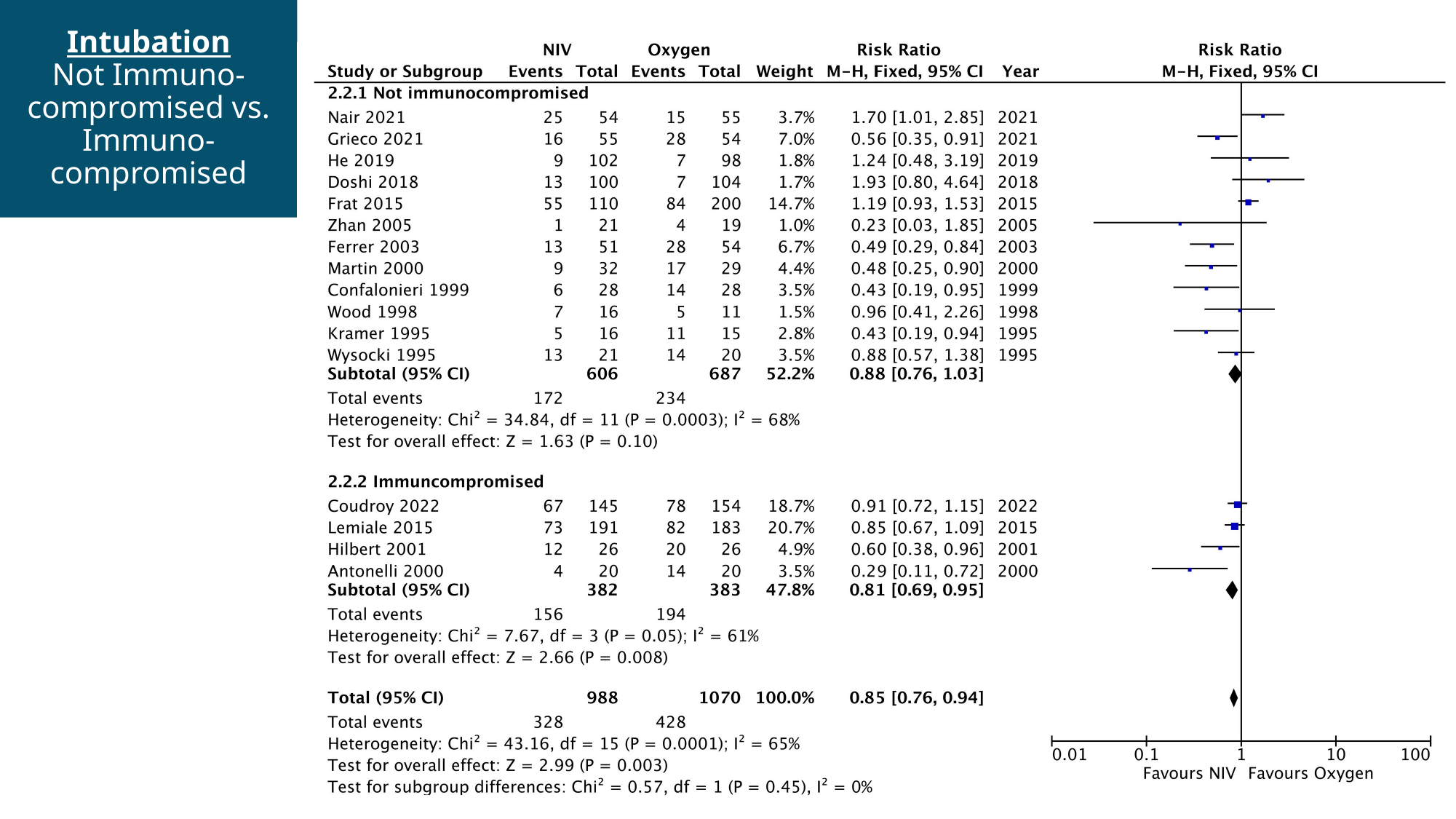

# Intubation Not Immuno-compromised vs. Immuno-compromised

Supplement: Supplementary file 7 — Supplementary material 7. [file 13613_2024_1367_MOESM7_ESM.pptx]

## Slide 1
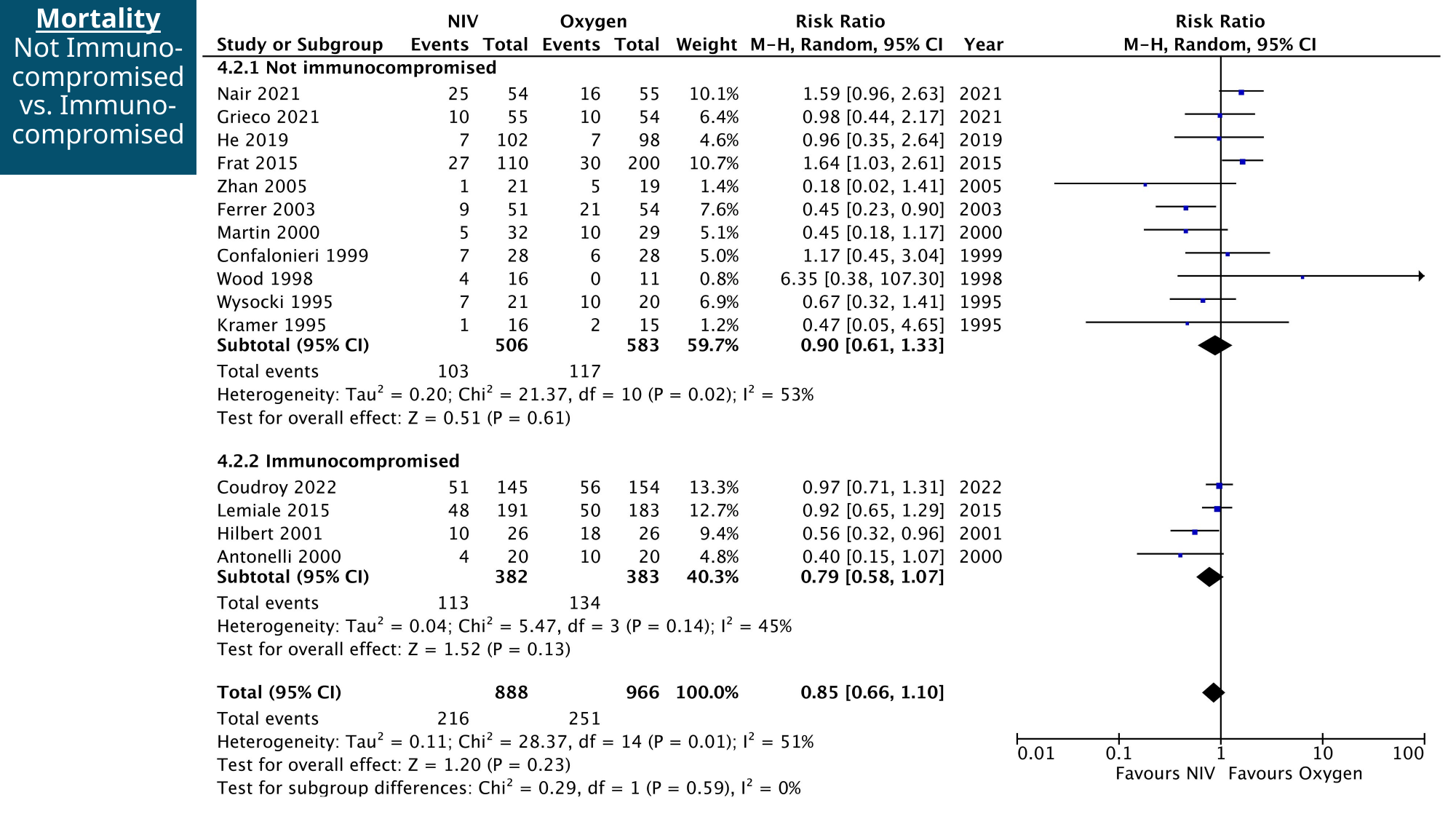

Mortality
Not Immuno-compromised vs. Immuno-compromised

Supplement: Supplementary file 8 — Supplementary material 8. [file 13613_2024_1367_MOESM8_ESM.pptx]
